# Phylogeny and taxonomic revisions of *Piper* (Piperaceae) in Taiwan

**DOI:** 10.1007/s10265-026-01733-0

**Published:** 2026-08-05

**Authors:** Po-Hao Chen, Chia-Hao Chang, Kuan-Ning Kung, Hung‑Chih Lin, An-Ching Chung, Chia-Ying Ou, Shih-Hui Liu

**Affiliations:** 1 Graduate Institute of Bioresources, National Pingtung University of Science and Technology, 1 Shuefu Rd., Neipu Township, Pingtung County, 91201 Taiwan; 2 Lioukuei Research Center, Taiwan Forest Research Institute, 198 Zhongzhuang, Chunghsing Village, Lioukuei Dist., Kaohsiung City, 844005 Taiwan; 3 Science Education, National Taipei University of Education, Taipei City, 106320 Taiwan; 4 Chiayi Research Center, Taiwan Forest Research Institute, Ln. 432, Wenhua Rd., West Dist., Chiayi City, 600054 Taiwan; 5 Division of Silviculture, Taiwan Forest Research Institute, 53, Nanhai Rd., Taipei, 100051 Taiwan; 6 Biological Sciences, National Sun Yat-Sen University, No.70 Lien-Hai Road, Kaohsiung City, 804201 Taiwan

**Keywords:** Huxley’s line, Molecular phylogeny, *Piper kadsura*, Taiwanese *Piper*, Wallace’s line

## Abstract

Taiwan, located at the northernmost extent of Huxley’s Line, marks the furthest point along this biogeographic boundary where exchanges of flora and fauna occur. The genus *Piper*, a globally distributed and taxonomically complex group of both economic and evolutionary importance, has a convoluted taxonomic history in this transitional zone that requires comprehensive study. Here, we integrate morphological, molecular, and distributional data to revise the taxonomy of Taiwanese *Piper*, resolve their evolutionary relationships, and infer their biogeographic history. Our revision recognizes 11 species in Taiwan, including seven native taxa, of which three are endemic. The present analysis establishes the first comprehensive phylogenetic framework for Taiwanese *Piper* and reveals that native species arose from lineages on both sides of Wallace’s Line. Collectively, these findings provide the first evidence of bidirectional expansion within a genus in Taiwan and position *Piper* as an exemplary system for examining phylogeographic dynamics in transitional biogeographic zones.

**Supplementary Information:**

The online version contains supplementary material available at https://doi.org/10.1007/s10265-026-01733-0.

## Introduction

*Piper* L. is an economically and evolutionarily important genus and the sixth-largest among flowering plants, comprising approximately 2,430 species primarily found in tropical regions worldwide (Jaramillo and Manos 2001; Moonlight et al. 2024; POWO 2025). Several *Piper* species, such as *P. magnificum* hort. ex Gentil, *P. nigrum* L., and *P*. *sarmentosum* Roxb., have significant economic value due to their applications in spices, ornamental horticulture, food preservation, and traditional medicine (Salehi et al. 2019; Sun et al. 2020; Takooree et al. 2019). Moreover, the remarkable diversity in morphology, floral biology, mating system, geographic distribution, and ecology makes *Piper* an excellent model for evolutionary studies (Dyer and Palmer 2004; Sen and Rengaian 2022). However, this same diversity also presents challenges to comprehensively understanding its taxonomy, reproductive biology, and evolutionary history (Frodin 2004; Moonlight et al. 2024; Sen and Rengaian 2022; Valentin-Silva 2023).

Previous phylogenetic studies have identified three major clades within *Piper*. Neotropics (ca. 1,300 spp.) occupy the basal branches, whereas Paleotropics—including the South Pacific (ca. 100 spp.) and Asian (ca. 600 spp.) clades—form a monophyletic group (Asmarayani 2018; Jaramillo et al. 2008; Jaramillo and Callejas 2004; Jaramillo and Manos 2001; Martínez et al. 2015; Metschina et al. 2025; Simmonds et al. 2021). Within the Asian clade, the West of Wallace’s Line (WWL) clade—which contains 12 subclades—is likely monophyletic and nested within the East of Wallace’s Line (EWL) clade, whose members are grouped into five subclades (Asmarayani 2018). Building on Asmarayani’s (2018) framework for Asian *Piper*, a few studies have investigated the evolutionary history and biogeography of selected taxa (Asmarayani 2023; Metschina et al. 2025; Sen et al. 2019; Sen and Rengaian 2022). However, the evolutionary relationships of many Asian *Piper* species remain poorly understood, and their taxonomy remains unresolved.

**Fig. 1:**
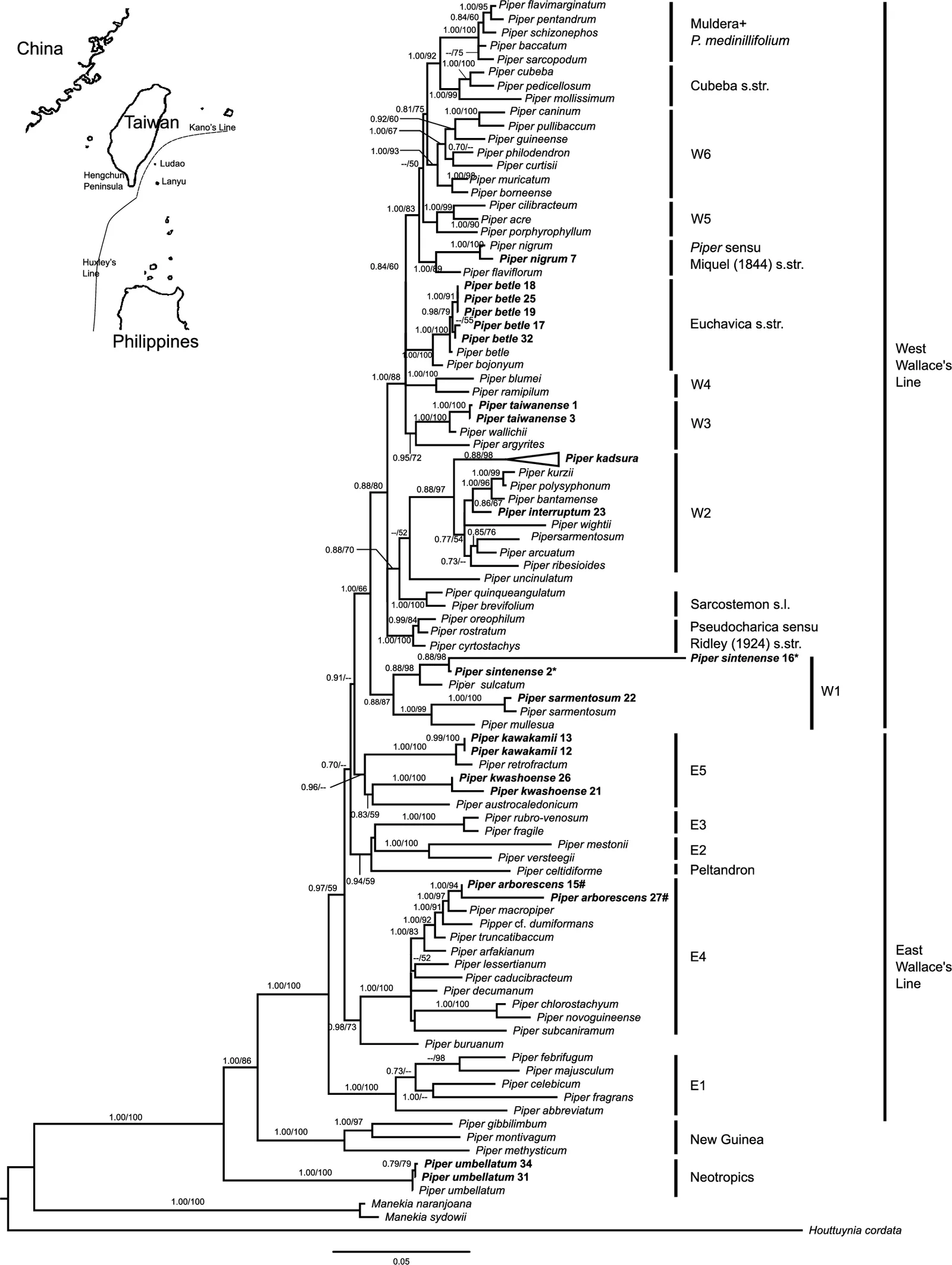
The map presents the distribution of Taiwan’s main island, Lanyu, Ludao, and the Hengchun Peninsula. The maximum likelihood tree illustrates the evolutionary relationships of Taiwanese *Piper* species. Vertical lines at the right denote the Asian clades. Numbers following species names correspond to the sample numbers listed in Table S1. Taxa marked with asterisks (*) and number signs (#) represent synonyms of *Piper laosanum* and *Piper macropiper*, respectively. Newly generated sequences are shown in bold. Posterior probabilities and bootstrap support values (> 70/ > 50%) are indicated at the corresponding branches

Taiwan (historically known as Formosa) is regarded as being located immediately west of the northernmost extent of Huxley’s Line, a hypothesized biogeographical boundary extending Wallace’s Line that delineates the distribution patterns of fauna and flora across Southeast Asia and the Pacific region (Ali and Heaney 2021; Huxley 1868; Simpson 1977; Wallace 1880) (Fig. 1). Together with the surrounding islands, Taiwan has a relatively young geological history, dating back to the Tertiary, compared to nearby landmasses and islands (Chai 1972; Ho 1986). Based on biogeographical data from various plant and animal groups, Kano proposed that the main island of Taiwan lies to the west of Huxley’s Line, while Lanyu (also known as Botel Tobago Island, Kôtôsho, or Orchid Island) and Ludao (Green Island, Kasyoto, Lyudao, or Kwashoto) are located to the east (Kano 1933, 1935a, b). The distinctive flora of the Hengchun Peninsula—the southernmost region of Taiwan’s main island—highlights its status as a ‘phytogeographical island,’ with plant composition more closely resembling that of Lanyu and Ludao than that of other areas on the main island (Li and Keng 1950). Numerous subsequent studies have supported or refined Kano’s Neo-Wallace Line (hereafter Kano’s Line) and the phytogeographical island concept (e.g., Hsu and Wolf 2009; Huang 2011; Kanehira 1935; Keng 1956; Liu and Ling 1978; Liu and Liu 1977; Masamune 1939; Tsai et al. 2015), emphasizing Taiwan’s significance for research on complex taxonomy and evolutionary history across these biogeographic regions.

To date, 11 species of *Piper* have been recognized in Taiwan, comprising four endemic species, three species also occurring in other areas, and four naturalized species (Chang and Kung 2020; Chung and Hsu 2017; de Candolle 1920; Editorial Committee of the Red List of Taiwan Plants 2017; Forbes and Hemsley 1891; Gardner 2006, 2010, 2013, 1917; Gilbert and Xia 1999; Hatusima 1935, 1970; Hayata 1908, 1911; Hayata and Matsumura 1906; Henry 1896; Hsu and Chung 2016; Kawakami 1910; Li 1963; Lin and Lu 1995, 1996; Liu et al. 1955; Liu and Wang 1976; Makino and Nemoto 1931; Masamune 1953, 1954; Ohwi 1934; Sasaki 1982; Tseng et al. 1999; Yamamoto 1932). The complex taxonomic history of Taiwanese *Piper* species is summarized in Table 1. This complexity may reflect a high rate of speciation or diversification in Taiwan and align with biogeographical expectations for Taiwan compared to other Asian regions, as suggested by many earlier studies focusing on diverse taxa (e.g., Hsieh 2002; Sun et al. 2025; Wallace 1880; Ye et al. 2023). In other words, Taiwanese *Piper* likely constitutes a valuable system for studying the phytogeographic history at the northernmost extent of Huxley’s Line.

**Table 1:** Taxonomic history and distributions of *Piper* species in Taiwan

Authors	Year	Taxa										
Forbes and Hemsley	1891	*P. futokadsura*	*P.* sp.									
Henry	1896	*P. futokadsura*	*P.* sp.	*P. betle*	*P. subpeltatum*							
Matsumura and Hayata	1906	*P. futokadsura*		*P. betle*	*P. subpeltatum*							
Hayata	1908	*P. futokadsura*										
Kawakami	1910	*P. futokadsura*	*P. subcordata*	*P. betle*	*P. subpeltatum*							
Hayata	1911		***P. kawakamii***		*P. subpeltatum*	***P. hispidum***	***P. kwashoense***					
Hayata	1917	*P. futokadsura*	*P. kawakamii & P. subcordata*		*P. subpeltatum*	*P. hispidum*	*P. kwashoense*					
Candolle	1920	***P. arboricola, P. subglaucescens**** & P. futokadsura*										
Sasaki	1928	*P. futokadsura*	*P. kawakamii & P. subcordata*	*P. betle*	*P. subpeltatum*	*P. hispidum*						
Makino and Nemoto	1931	*P. futokadsura*	*P. kawakamii*		*P. subpeltatum*	*P. hispidum*	*P. kwashoense*					
Yamamoto	1932							***P. kotoense***				
Ohwi	1934	*P. futokadsura*										
Hatusima	1935	*P. kadsura*				***P. sintenense***						
Masamune	1953											
Masamune	1954	*P. futokadsura*	*P. kawakamii & P. subcordata*		*P. subpeltatum*	*P. hispidum & P. sintenense*		*P. kotoense*				
Liu et al	1955	*P. kadsura*	*P. kawakamii & P. subcordata*		*P. umbellatum* var.* subpeltatum*		*P. philippinum*	*P. arborescens var. angustilimbum*	*P. interruptum*			
Li	1963		*P. kawakamii*		*P. subpeltatum*	*P. arboricola*						
Hatusima	1970	*P. futokadsura*		*P. betle*			*P. philippinum*	*P. arborescens var. angustilimbum*	*P. interruptum* var. *multinervum*			
Liu and Wang	1976		*P. kawakamii*	*P. betle*	*P. subpeltatum*	*P. arboricola*	*P. kwashoense & P. philippinum*	*P. arborescens*	*P. interruptum*			
Lin and Lu	1995	*P. kadsura*								***P. taiwanense***		
Lin and Lu	1996		*P. kawakamii*	*P. betle*	*P. umbellatum*	*P. sintenense*	*P. philippinum*	*P. arborescens*	*P. interruptum* var. *multinervum*	*P. taiwanense*		
Gilbert and Xia	1999	*P. kadsura*				*P. sintenense*	*P. kwashoense*					
Tseng et al	1999	*P. kadsura*	*P. kawakamii*		*P. umbellatum*	*P. sintenense*	*P. kwashoense*	*P. arborescens*	*P. interruptum*	*P. taiwanense*		
Gardner	2006	*P. kadsura*					*P. philippinum*	*P. macropiper*	*P. interruptum*			
Gardner	2010							*P. macropiper*	*P. interruptum*			
Gardner	2013						*P. insectifugum*	*P. macropiper*	*P. interruptum*			
Hsu and Chung	2016										*P. sarmentosum*	
Chung and Hsu	2017		*P. kawakamii*	*P. betle*	*P. umbellatum*	*P. sintenense*	*P. philippinum*	*P. arborescens*	*P. interruptum* var.* multinervum*	*P. taiwanense*	*P. sarmentosum*	*P. aduncum*
Editorial Committee of the Red List of Taiwan Plants	2017	*P. kadsura*	*P. kawakamii*	*P. betle*	*P. umbellatum*	*P. sintenense*	*P. kwashoense*	*P. arborescens*	*P. interruptum* var.* multinervum*	*P. taiwanense*	*P. sarmentosum*	
Chang and Kung	2020	*P. kadsura*		*P. betle & P. kwashoense*			***P. lanyuense***	*P. arborescens*	*P. interruptum*			
This study		*P. kadsura*	*P. kawakamii*	*P. betle*	*P. umbellatum*	*P. laosanum*	*P. kwashoense*	*P. macropiper*	*P. interruptum*	*P. taiwanense*	*P. sarmentosum*	*P. aduncum*
Distributions of the taxon		China, Japan, Korea, Taiwan (excluding Lanyu), Vietnam	Southern Taiwan including Hengchun Peninsula (excluding Lanyu and Ludao) (endemic to Taiwan)	Naturalized	Naturalized	Laos, Taiwan (excluding Lanyu and Ludao), Thailand	Lanyu, Ludao (endemic to Taiwan)	Australia, Futuna Is., India, Indonesia, Malaysia, Micronesia, Philippines, Samoa,Taiwan (Lanyu only), Vanuatu	Papuasia, Philippines, Queensland, Taiwan (Lanyu only), Vanuatu	Taiwan (excluding Lanyu and Ludao) (endemic to Taiwan)	Naturalized	Naturalized

Type specimens originating from Taiwan are highlighted in bold

According to earlier phylogenetic studies of Asian *Piper* (Asmarayani 2018; Sen et al. 2019), *P. kadsura* (Choisy) Ohwi—which occurs in Taiwan, Korea, and Japan—belongs to the W2 clade. However, the phylogenetic positions of other Taiwanese *Piper* species remain unresolved. It is unclear whether those in the Hengchun Peninsula, Lanyu, and Ludao originated from the EWL clades, and whether species in other regions of Taiwan derived from the WWL clades. A comprehensive phylogeny including all Taiwanese *Piper* species would be needed to understand their evolutionary history and diversification.

In addition, a thorough reassessment of the complex taxonomy of Taiwanese *Piper* is clearly warranted. In earlier taxonomic literature (Table 1), *P. kwashoense* Hayata has been referred to by three other names––*P. insectifugum* C.DC., *P. lanyuense* K.N.Kung and Kun C.Chang, and *P. philippinum* Miq.. Similarly, *P. sintenense* Hatusima has been identified under other names, including *P. arboricola* C.DC., *P. hispidum* Hayata, and *P. laosanum* C.DC (Table 1). In addition, *P. arborescens* Roxb. has been recorded under other names, including *P. arborescens* var. *angustilimbum* Quis, *P. kotoense* Yamam, and *P. macropiper* Pennant (Table 1)*. Piper kadsura* (hereafter referred to as the *Piper kadsura* complex) has likewise been described under other names, including *P. arboricola*, *P. futokadsura* Sieb., and *P. subglaucescens* C.DC. (Table 1). Although *P. kadsura* has been widely adopted in recent systematic literature (Chung and Hsu 2017; Editorial Committee of the Red List of Taiwan Plants 2017; Gilbert and Xia 1999; Lin and Lu 1996; Liu and Wang 1976; Masamune 1954; Tseng et al. 1999), *P. arboricola* is still retained in a few studies (de Candolle 1920; Jaramillo and Callejas 2004). Moreover, this complex appears to have also been documented from some localities in mainland China (Li et al. 2025; Tseng et al. 1999) and may represent a form of *P. wallichii* (Miquel) Handel-Mazzetti (Tseng et al. 1999). These nomenclatural inconsistencies highlight the necessity for an integrative approach combining morphological and molecular evidence to resolve longstanding taxonomic ambiguities within the group.

In the present study, we aimed to incorporate morphological characteristics, molecular data, and distribution information to (1) revise the taxonomy of the genus in Taiwan and (2) investigate the evolutionary history of Taiwanese *Piper* spanning the northernmost extent of Huxley’s Line*.*

## Materials and methods

### Herbarium specimen investigation

To assess the morphological characteristics of Taiwanese *Piper*, an exhaustive investigation of specimens from B, CHIA, HAST, K, MO, NCKU, NTUF, P, PPI, TAI, TAIF, TCF, TI, and TNM herbaria (Thiers 2016) was carried out. Moreover, field observations and sample collection were conducted in Taiwan, Japan, and Hong Kong to study phenological phases and floral morphology and to reconstruct molecular phylogeny. The collected samples were then transplanted to the greenhouse at the Lioukuei Research Center, Taiwan, for closer observation.

### Morphological character scoring and phenetic analyses

To summarize overall morphological similarity patterns among Taiwanese *Piper* species and facilitate comparison with molecular phylogenies, we conducted a distance-based phenetic analysis of morphological characters. We included all 11 *Piper* species occurring in Taiwan and scored three specimens per species as operational taxonomic units (OTUs), yielding 33 OTUs (OTU 1–33, Table 2). Morphological characters of each OTU were compiled as a mixed dataset consisting of quantitative traits (measured in millimeters) and qualitative traits. Quantitative traits comprised mean leaf length, mean leaf width, leaf length/width ratio, and mean petiole length. Qualitative traits––including venation type, inflorescence orientation, leaf texture, habit, leaf shape, leaf base, vein-number categories, leaf-base asymmetry, and indumentum––were encoded as binary (0/1) dummy variables.

**Table 2:** List of operational taxonomic units (OTUs) examined in the morphological phenetic analysis of Taiwanese *Piper*. Three vouchers were used for each of the 11 Taiwanese *Piper* species (OTU1–OTU33). For each OTU, the collector and collection number, together with the herbarium code, are provided

Species	OTU	Voucher (collector and number)	Herbarium
*Piper aduncum* L	OTU1	S.M. Ku 10	PPI
*Piper aduncum* L	OTU2	S.Z. Yang 27,897	PPI
*Piper aduncum* L	OTU3	P.H. Chen 1311	TAIF
*Piper betle* L	OTU4	P.H. Chen 2205	PPI
*Piper betle* L	OTU5	P.H. Chen 446	PPI
*Piper betle* L	OTU6	P.H. Chen 451	PPI
*Piper interruptum* Opiz	OTU7	C.E. Chang 22,097	PPI
*Piper interruptum* Opiz	OTU8	T.C. Hsu 3898	TAIF
*Piper interruptum* Opiz	OTU9	W.Y. Wang 3036	TAIF
*Piper kawakamii* Hayata	OTU10	C.L. Yeh s.n	PPI
*Piper kawakamii* Hayata	OTU11	S.Z. Yang 25,087	PPI
*Piper kawakamii* Hayata	OTU12	M.Y. Shen 4748	TAIE
*Piper kwashoense* Hayata	OTU13	P.H. Chen 448	PPI
*Piper kwashoense* Hayata	OTU14	P.H. Chen 2218	PPI
*Piper kwashoense* Hayata	OTU15	G.P. Hsieh 2083	PPI
*Piper laosanum* C.DC	OTU16	P.H. Chen 695	PPI
*Piper laosanum* C.DC	OTU17	P.H. Chen 696	PPI
*Piper laosanum* C.DC	OTU18	P.H. Chen 2021	PPI
*Piper macropiper* Pennant	OTU19	P.H. Chen 583	PPI
*Piper macropiper* Pennant	OTU20	P.H. Chen 2197	PPI
*Piper macropiper* Pennant	OTU21	C.E. Chang 19,217–2	PPI
*Piper sarmentosum* Roxb	OTU22	K.W. Li 169	PPI
*Piper sarmentosum* Roxb	OTU23	S.Z. Yang 26,698	PPI
*Piper sarmentosum* Roxb	OTU24	P.H. Chen 1329	TAIF
*Piper kadsura* (Choisy) Ohwi	OTU25	P.H. Chen 203	PPI
*Piper kadsura* (Choisy) Ohwi	OTU26	P.H. Chen 659	PPI
*Piper kadsura* (Choisy) Ohwi	OTU27	P.H. Chen 660	PPI
*Piper taiwanense* Lin & Lu	OTU28	P.H. Chen 396	PPI
*Piper taiwanense* Lin & Lu	OTU29	P.H. Chen 694	PPI
*Piper taiwanense* Lin & Lu	OTU30	P.H. Chen 2242	PPI
*Piper umbellatum* L	OTU31	X.N. Bai 97	PPI
*Piper umbellatum* L	OTU32	P.H. Chen 2464	PPI
*Piper umbellatum* L	OTU33	T.Y.A. Yang et al. 5930	PPI

Because the dataset comprised both quantitative traits and binary-coded qualitative variables, pairwise dissimilarities among OTUs were calculated using the Gower distance coefficient Gower JC (1971). Hierarchical clustering was performed using UPGMA (unweighted pair-group method with arithmetic mean) in PAST v5.2 (Hammer et al. 2001) to generate a dendrogram (phenogram) summarizing overall morphological similarity among OTUs.

### DNA sample collection, isolation, marker amplification, and sequencing

To provide a comprehensive understanding of the phylogeny and origins of *Piper* in Taiwan, we intended to collect one to two samples for each Taiwanese *Piper* taxon from the field. Additionally, multiple samples of the *P. kadsura* complex from different localities were obtained to explore the evolutionary relationships within the complex. Both specimen vouchers and young leaf materials for molecular analyses were collected. The specimen vouchers were deposited at PPI, TAIE, TAIF, and TNM herbaria (Thiers 2016) for further investigation, with detailed voucher information provided in Table S1. Newly collected young leaf materials were immediately dried in silica gel after collection.

Genomic DNA from *Piper* samples was isolated using the Plant Genomic DNA Extraction Miniprep System (Viogene, Taipei, Taiwan), following the manufacturer’s protocol. The isolated genomic DNA was then validated using 1.0% agarose gel electrophoresis. To incorporate as many Asian *Piper* taxa as possible in our phylogenetic analysis and to minimize potential bias caused by missing data (e.g., Brinkmann et al. 2005; Stefanović et al. 2004; Wiens 2005), we selected ITS (comprising ITS1, the 5.8 s gene, and ITS2) and *trn*L-F (including the tRNA-Leu gene and the intergenic spacer *trn*L-*trn*F) as DNA markers, both of which are widely used in previous *Piper* systematic studies (e.g., Asmarayani 2018; Jaramillo et al. 2008; Jaramillo and Callejas 2004; Jaramillo and Manos 2001; Martínez et al. 2015; Sen et al. 2019; Simmonds et al. 2021). Following amplification with universal primers (Taberlet et al. 1991; White et al. 1990), the products were examined via 1.0% agarose gel electrophoresis. The primers and optimal amplification conditions are provided in Table S2. The validated amplified products were then purified using the QIAquick PCR Purification kit (Qiagen, Hilden, Germany) and commercially sequenced on an ABI 3730XL platform at Genomics BioSci & Tech Co., Ltd., Taipei, Taiwan, using the primers listed in Table S2.

Published ITS and *trn*L-F sequences of other Asian *Piper* taxa were downloaded from GenBank (Sayers et al. 2019). Additionally, sequences of *Manekia naranjoana* (C.DC.) Callejas ex N. Zamora, Hammel and Grayum, *M. sydowii* (Trel.) T. Arias, Callejas and Bornst., *Houttuynia cordata* Thunb., *Peperomia pellucida* (L.) Kunth, and *Peperomia serpens* (Sw.) Loudon were obtained from GenBank (Sayers et al. 2019) to represent outgroup genera. A complete list of taxa included in this study, along with their accession numbers, is provided in Table S1.

### Phylogenetic analyses

Newly generated DNA reads were assembled using the Geneious De Novo Assemble tool in Geneious Prime 2025.0 (https://www.geneious.com) and aligned with published sequences using MUSCLE 5.1 (Edgar 2022) embedded in Geneious Prime 2025.0. The best-fit nucleotide substitution models for the ITS, *trn*L-F, and concatenated alignments were evaluated using jModeltest 2.1.10 (Darriba et al. 2012). Maximum likelihood (ML) and Bayesian inference (BI) trees based on ITS, *trn*L-F, and concatenated alignments were generated separately using RAxML-VI-HPC (Stamatakis 2006) in Geneious Prime 2022.1 and MrBayes 3.2.7a (Ronquist et al. 2012; Ronquist and Huelsenbeck 2003) in the CIPRES Science Gateway (Miller et al. 2010), respectively. Bootstrap support (bs) for ML trees was estimated with 1,000 replicates. Each BI tree was constructed from two independent runs of 5 × 10^6^ generations, with posterior probabilities (pp) summarized after applying a 0.25 burn-in. The Shimodaira-Hasegawa (SH) and Kishino-Hasegawa (KH) tests (Kishino and Hasegawa 1989) in PAUP* 4.0a (Swofford 2003) were used to assess topology conflict between ITS and *trn*L-F trees. Finally, FigTree 1.4.4 (Miller et al. 2010) was employed to visualize the trees.

To explore interspecific relationships within the *P. kadsura* complex, an additional ITS tree was reconstructed using newly generated sequences along with published sequences of *P. arboricola* and *P. kadsura* from GenBank (Sayers et al. 2019). Moreover, our preliminary phylogenetic analysis indicates that *P. hancei* Maximowicz clusters closely with *P. kadsura*. Therefore, additional published sequences of *P. hancei* from GenBank were also included in the analysis. Sample information for the published ITS sequences of the complex is summarized in Table S3.

## Results

### Phenetic analyses

**Fig. 2:**
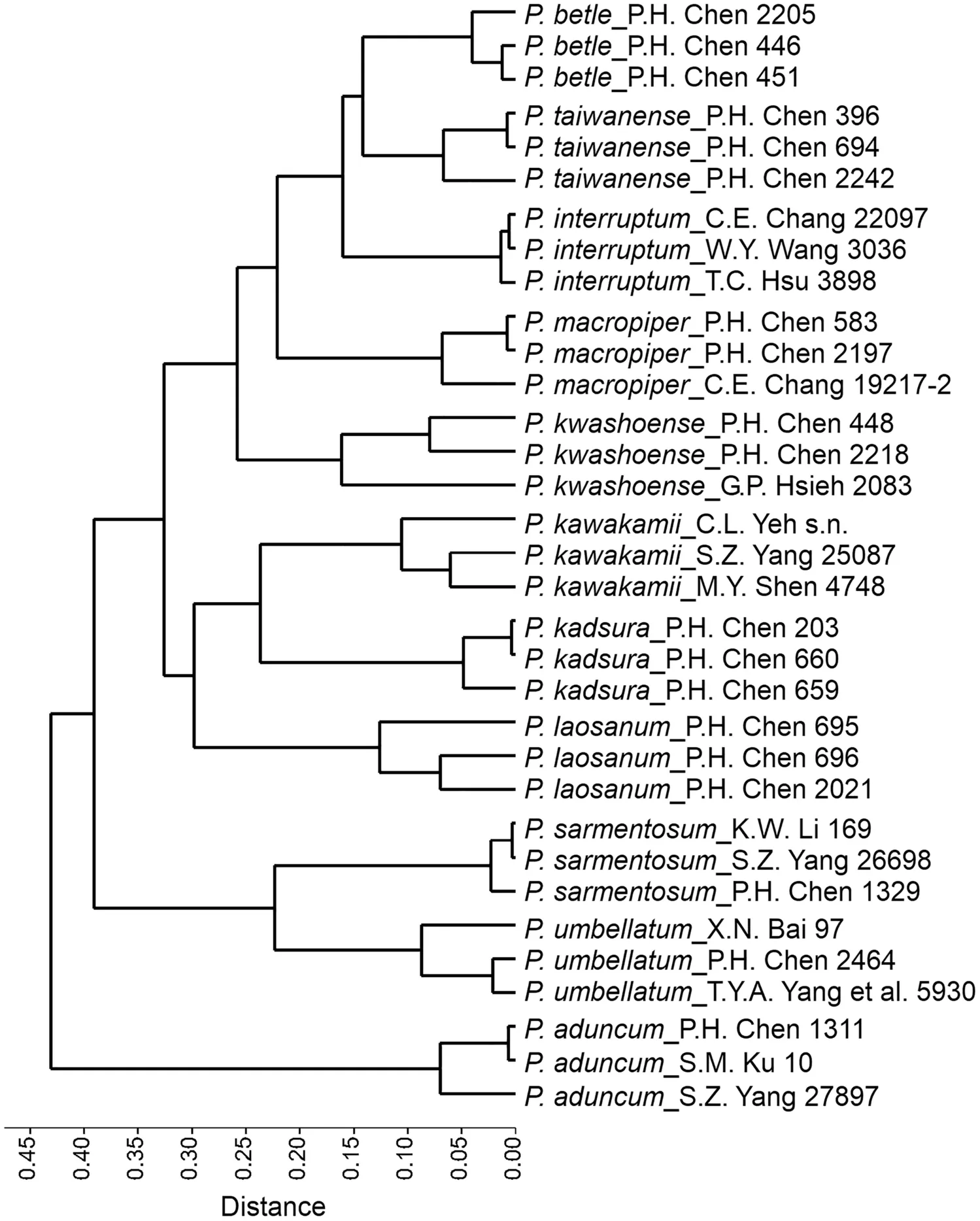
The phenogram depicts morphological similarity among 11 Taiwanese *Piper* with three OTUs representing each taxon. Details of the specimen vouchers are provided in Table 2

Our phenetic analysis based on 33 OTUs representing all 11 Taiwanese *Piper* species suggests that conspecific OTUs clustered tightly and merged at low dissimilarity values (typically < 0.1), indicating strong within-species morphological cohesion across the characters scored (Fig. 2 and Data S1).

### Phylogenetic analyses

In total, 303 sequences representing 193 accessions, including 108 *Piper* taxa and five outgroups, were applied in our phylogenetic analyses. Seventy-six sequences from 38 *Piper* accessions are newly generated. These newly collected samples represent all Taiwanese *Piper* taxa, except for *P. aduncum* L., a naturalized *Piper* species from the Americas. The scientific names, GenBank accession numbers, and voucher information for the newly collected samples are available in Table S1. The alignment statistics are summarized in Table 3.

**Table 3:** The total number of accessions, the number of *Piper* accessions, the number of *Piper* taxa, the number of outgroups, sequence lengths, the number of parsimony-informative characters, and the best-fit nucleotide substitution models for the alignments studied in this research

Alignment	Total # of accessions	# of *Piper* accessions	# of *Piper* taxa	# of outgroups	Length of alignment	# of parsimony-informative characters	The best-fit nucleotide substitution model
ITS	146	141	105	5	872	421	TPM1uf+G
*trn*L-F	106	103	75	3	944	115	TPM1uf+G
Concatenated (ITS + *trn*L-F)	111	108	78	3	1,813	485	TVMef+I+G
*Piper kadsura* complex (ITS)	72	72	12	0	678	191	TIM3+G

Both KH and SH tests revealed significant differences between the *trn*L-F and ITS tree topologies (Table S4a), and the former tree exhibits generally low resolution. The *trn*L-F and ITS alignments and trees are provided in Figs. S1–S2 and Data S2–S3. To achieve higher tree resolution, we employed a concatenated alignment to reconstruct the final BI and ML trees (Fig. 1 and Data S4), as recommended in previous phylogenetic studies (Burleigh and Mathews 2004; Kupczok et al. 2010; Soltis et al. 1998). Since the BI and ML trees are topologically congruent (*p* > 0.05 in both KH and SH tests; Table S4b), only the ML tree is presented here (Fig. 1).

Our phylogenetic tree is largely consistent with previous studies (Asmarayani 2018; Metschina et al. 2025; Sen et al. 2019; Simmonds et al. 2021). This analysis represents the first comprehensive phylogeny of Taiwanese *Piper* and reveals that these taxa are distributed across the West Wallace’s Line, East Wallace’s Line, and Neotropics clades (Fig. 1). As expected, the naturalized *P. betle* L. and *P. sarmentosum* are clustered with taxa in the Euchavica s.str. and W1 clades, respectively. *Piper umbellatum* L. is grouped with Neotropics clades. Moreover, our study presents the phylogenetic placements of *P. interruptum* Opiz, *P. kawakamii* Hayata, *P. kwashoense*, and *P. taiwanense* Lin and Lu for the first time.

Unexpectedly, *P. arborescens* does not appear as a monophyletic taxon and clusters with *P. macropiper* in the E4 clade instead (Figs. 1, S2). Similarly, *P. sintenense* samples do not form a monophyletic group but group with *P. laosanum* in the W1 clade (Figs. 1, S2). These results highlight the need for taxonomic revisions.

In addition, accessions within the *P. kadsura* complex form a well-supported monophyletic group (pp = 0.88, bs = 98) nested within the W2 clade. Therefore, a taxonomic revision of the *P. kadsura* complex is provided here.

### Interspecific relationships within the *P. kadsura* complex

**Fig. 3:**
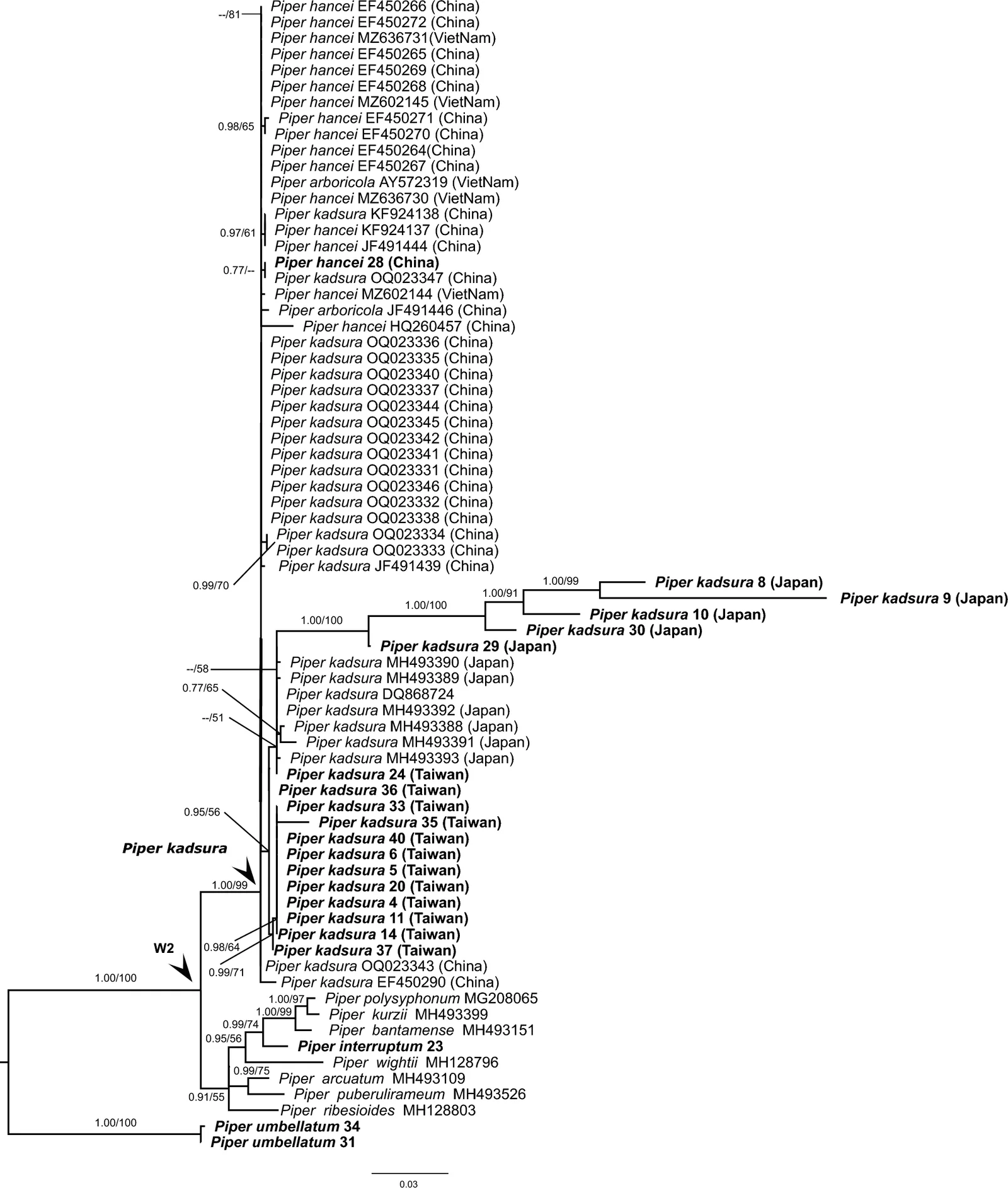
The maximum likelihood tree illustrates the evolutionary relationships among samples within the *P. kadsura* complex. Numbers following scientific names indicate the sample number of newly generated sequences listed in Table S1. GenBank accession numbers following species names refer to sequences obtained from GenBank. Posterior probabilities and bootstrap support values (> 70/ > 50%) are indicated at the corresponding branches. The arrow labeled ‘W2’ indicates the crown node of the W2 clade as shown in Fig. 1, while the arrow labeled ‘*Piper kadsura*’ marks the monophyly of the *P. kadsura* complex

A total of 72 ITS sequences––including 62 accessions from the *P. kadsura* complex, eight other *Piper* taxa from the W2 clade, and two accessions of *P. umbellatum* from the Neotropics clade––were applied to reconstruct the *P. kadsura* complex ML and BI trees (Data S5). Only the ML tree is shown here (Fig. 3). The *P. kadsura* complex tree indicates that *P. arboricola*, *P. hancei*, and *P. kadsura* are interwoven within a well-supported clade (pp = 1.00, bs = 99), with none forming a monophyletic clade. That is to say, *P. arboricola*, *P. hancei*, and *P. kadsura* are conspecific.

### Taxonomic treatments

***Piper*** L., Sp. Pl. 1:28. 1753.

Through an integrative analysis of morphological and molecular evidence, we propose an updated taxonomic treatment for the 11 *Piper* taxa currently recognized in Taiwan.

### Key to *Piper* of Taiwan

1. Trees, lamina elliptic, leaves venation pinnate with more than 5 pairs of secondary veins 

… *P. aduncum*

-1. Herbs, shrubs, or lianas; leaves venation palmate or pinnate with fewer than 5 pairs of secondary veins.

2. Herbs or shrubs, lamina cordate.

3. Prostrate herb; inflorescences solitary, flowers unisexual; leaves glabrous

… *P. sarmentosum*

*.* -3. Small shrub; inflorescences umbellate, flowers bisexual; leaves pubescent

 … *P. umbellatum.*

-2. Lianas; lamina ovate to ovate-elliptic, ovate-lanceolate, elliptic to elliptic-lanceolate, rarely cordate.

4. Venation pinnate; leaf base usually asymmetrical.

5. Secondary veins 3; leaf surface pubescent; inflorescences pendulous 

… *P. laosanum.*

-5. Secondary veins 2 or 4; leaf surface glabrous; inflorescences erect.

6. Leaves thinly fleshy; secondary veins 2; leaf base acute 

… *P. kadsura*

-6. Leaves papery; secondary veins 4; leaf base rounded to cordate. 

… *P. kawakamii.*

-4. Venation palmate; leaf base usually symmetrical.

7. Leaves coriaceous; inflorescences erect

 … *P. kwashoense*

-7. Leaves papery to thinly fleshy; inflorescences pendulous.

8. Leaves papery; lamina ovate-lanceolate; leaf base acute or rounded 

… *P. macropiper*

-8. Leaves thinly fleshy; lamina ovate to ovate-elliptic or ovate-lanceolate; leaf base cordate or rounded.

9. Basal veins 7; leaf base rounded 

… *P. interruptum*

-9. Basal veins 3 or 5; leaf base cordate.

10. Lamina usually smaller (length < 10 cm, width < 5 cm); petiole shorter (< 1.5 cm); basal veins 3 

… *P. taiwanense*

-10. Lamina usually larger (length > 10 cm, width > 5 cm); petiole longer (> 1.5 cm); basal veins 5 

… *P. betle.*

**Fig. 4:**
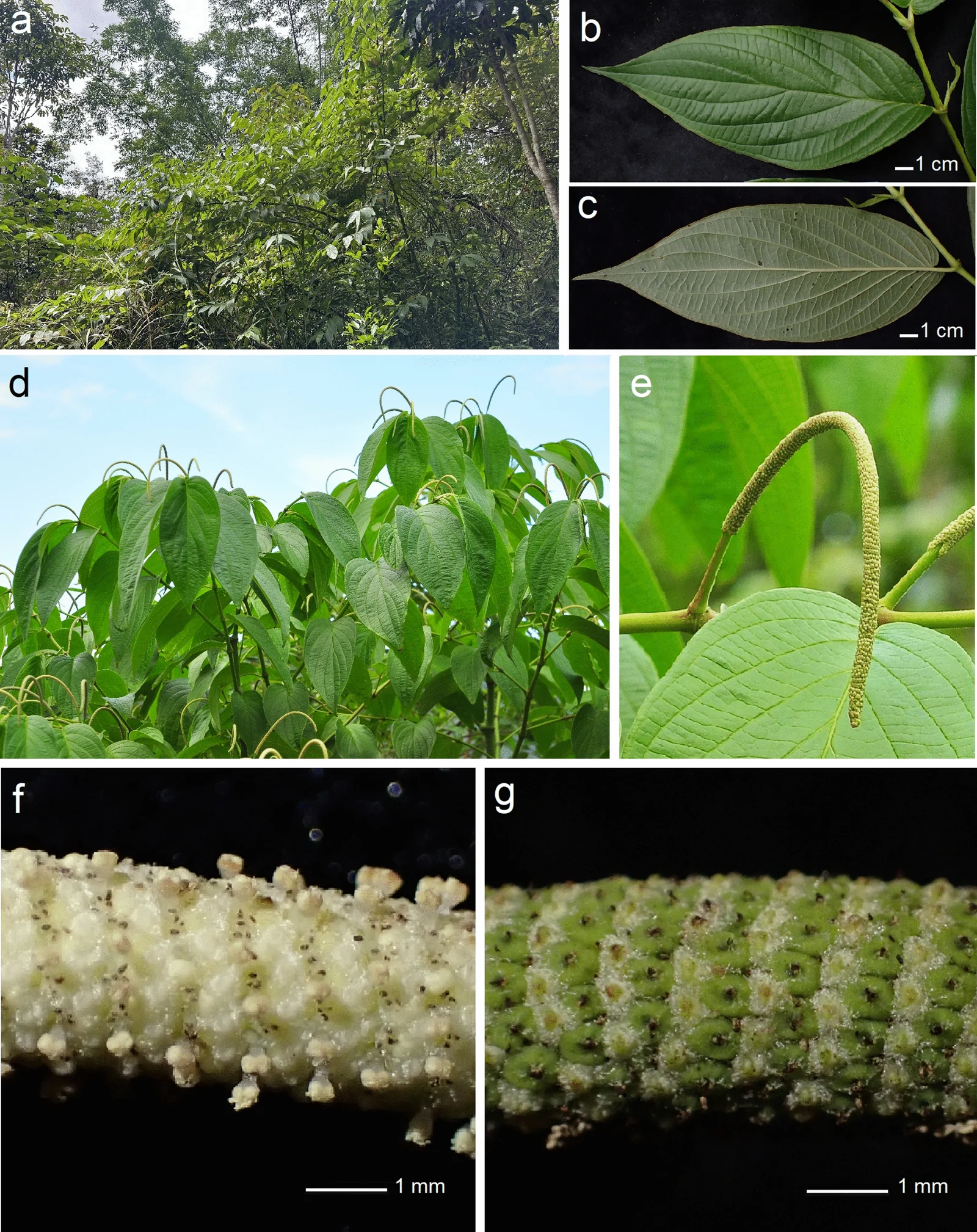
*P. aduncum* : **a** habit; **b** adaxial surface of gonophyll leaf; **c** abaxial surface of gonophyll leaf; **d** branch with inflorescence; **e** inflorescence; **f** a portion of inflorescence; **g** a portion of infructescence. All photographs by Po-Hao Chen

***Piper aduncum*** L. Sp. Pl. 29. 1753; Chung and Hsu, Illustr. Fl. Taiwan. 1: 129, 2017. **Type**: Plumier, Descr. Pl. Amér., 53, t. 77 1693 (lecto. designated by H. Saralegui Boza 2004). (Figs. 4, 15a).

Diagnosis: *P. aduncum* is the only arborescent (tree) species of *Piper* in Taiwan. It is characterized by elliptic to elliptic-lanceolate leaves with pinnate venation bearing more than five pairs of secondary veins, and erect inflorescences (often arching or recurved), and it is bisexual**.** Although bisexual flowers also occur in *P. umbellatum*, *P. aduncum* is readily distinguished from that species by its elliptic leaves with pinnate venation**,** whereas *P. umbellatum* has cordate leaves with palmate venation**.**

Distribution: Taiwan: Naturalized. The species is sporadically distributed in low-elevation mountainous regions of northern and central Taiwan (Chung and Hsu 2017).

Native Range: Tropical America (Burger 1971; Hartemink 2010).

Phenology: Flowering: all year.

Conservation status: *P. aduncum* was not assessed in the Editorial Committee of the Red List of Taiwan Plants (2017); given its status as a naturalized species in Taiwan (Chung and Hsu 2017), it is treated here as Not Applicable (NA)**.**

Specimens examined: TAIWAN. Taipei: Xindian, *P.H. Chen 1311* (TAIF). Nantou: Lienhuachih, *Y.C. Hsieh YCH0233* (TAIF).

Notes: *P. aduncum* is a naturalized species in Taiwan (Chung and Hsu 2017) and is readily distinguishable from other Taiwanese *Piper*.

**Fig. 5:**
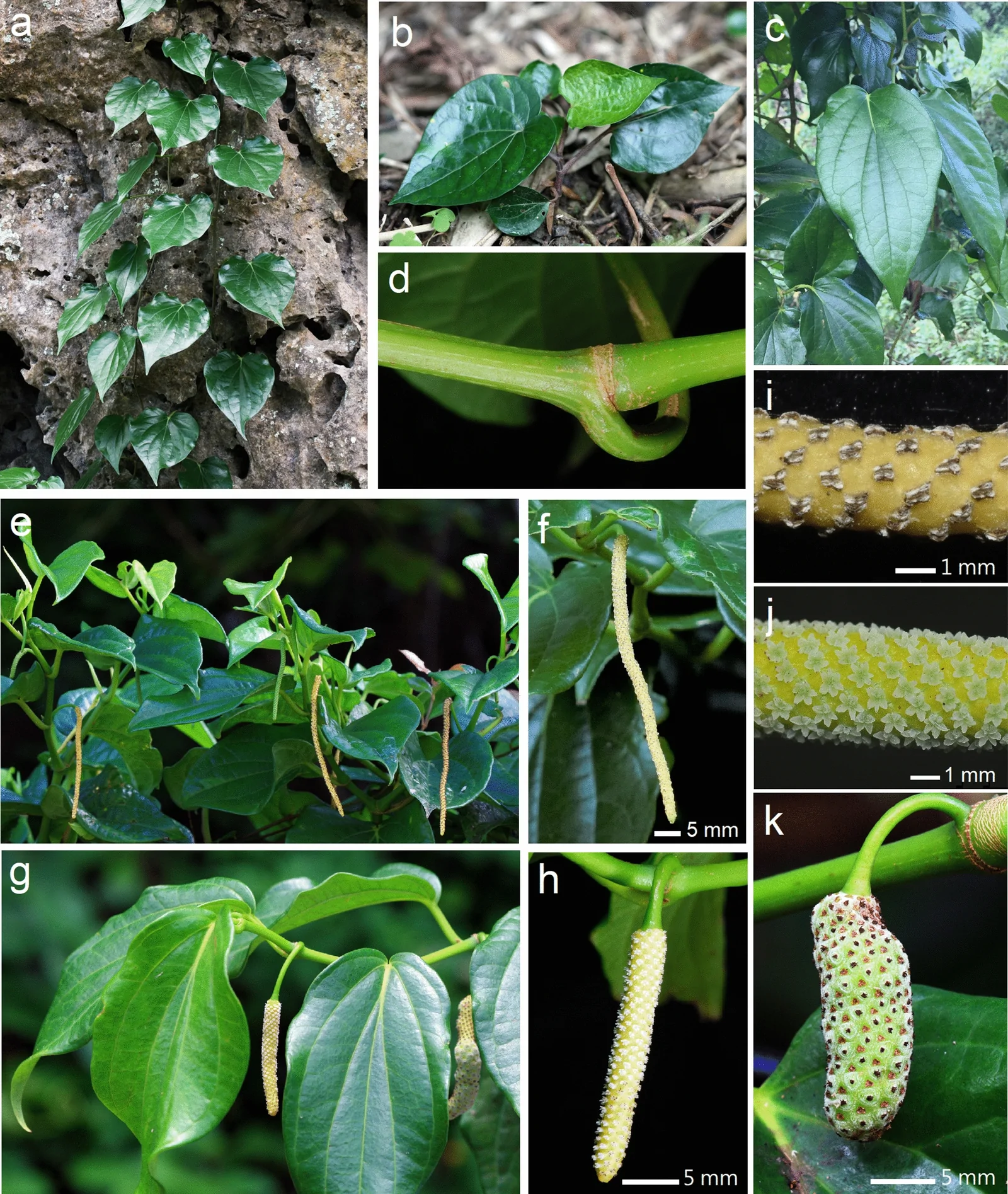
*P. betle* : **a** habit; **b** young leaves; **c** gonophyll leaf; **d** branch; **e** branch with male inflorescence; **f** male inflorescence; **g** branch with female inflorescence;** h** female inflorescence; **i** a portion of male inflorescence; **j** a portion of female inflorescence; **k** infructescence. Photographed by Kuan-Ning Kung (**a**, **b**, **d**–**h**, **k**), and Po-Hao Chen (**c**, **i**, **j**)

***Piper betle*** L., Sp. Pl. 28. 1753; Henry, List Pl. Form. 77. 1896; Kawakami, List Pl. Form. 94. 1910; Sasaki, List Pl. Form. 141. 1928; Masamune, Short Fl. Form. 38. 1936; Hatusima, Mem. Fac. Agr. 7(1): 302. 1969; Liu and Wang in Fl. Taiwan 2: 561. 1976; Liu et al., Tre. Taiwan 798. 1988; Tre. Taiwan rev. ed. 695. 1994; Lin and Lu, Fl. Taiwan 2^nd^ed. 2: 628. 1996; Tseng et al*.*, Fl. China 4: 130. 1999; Chung and Hsu, Illustr. Fl. Taiwan. 1: 130, 2017. **Type**: Sri Lanka, *Herb. Hermann 3: 32, No. 27* (lecto. BM000621919 designated by H. Huber 1987). (Figs. 5, 15b).

Diagnosis: *P. betle* is characterized by ovate to ovate-elliptic leaves, a cordate leaf base**,** venation palmate with five primary veins, and pendulous inflorescences. It is readily distinguished from *P. taiwanense* by having five (vs. three) primary veins, and from *P. interruptum* by its cordate (vs. rounded) leaf base and the absence of a seven-veined palmate pattern.

Distribution: Taiwan: Naturalized. The species is distributed in the low-elevation areas of central, southern, and eastern Taiwan, with larger populations found on the offshore islands of Lanyu and Ludao.

Native Range: Indo-China to Malesia (Biswas et al. 2022; POWO 2025; Zumbroich 2008).

Phenology: Flowering: April to August. Fruiting: June to October.

Conservation status: Taiwan Red List category: Not Applicable (NA) (Editorial Committee of the Red List of Taiwan Plants 2017). *P. betle* is treated as a naturalized species in Taiwan and is therefore listed as NA in the Red List.

Specimens examined (representative). TAIWAN. Changhua: Tianzhong, Pengshan Temple, *S.F. Huang 4942* (TAI). Nantou: Lingxiaodian, *P.H. Chen 1273* (TAIF). Chiayi: Lantan, *Kung et Ku 166* (CHIA). Kaohsiung: Shanping, *S.Z. Yang 42,081* (PPI). Pingtung: Neipu, *P.H. Chen 3712* (TAIE); Neiwen, *P.H. Chen 3713* (TAIE). Taitung: Lanyu Township, *P.H. Chen 3726* (TAIE); Lanyu Township, *C.C. Hsu 12,284* (TAI); Ludao Township, *C.H. Chen *et al*. 7096* (TNM); Wenquan Village, Ludao Township, Guoshan Trail, *G.P. Hsieh 1917* (PPI). Additional specimens examined are listed in Data S6.

Notes: *P. betle* is a naturalized species in Taiwan and is readily distinguishable from other Taiwanese *Piper*.

**Fig. 6:**
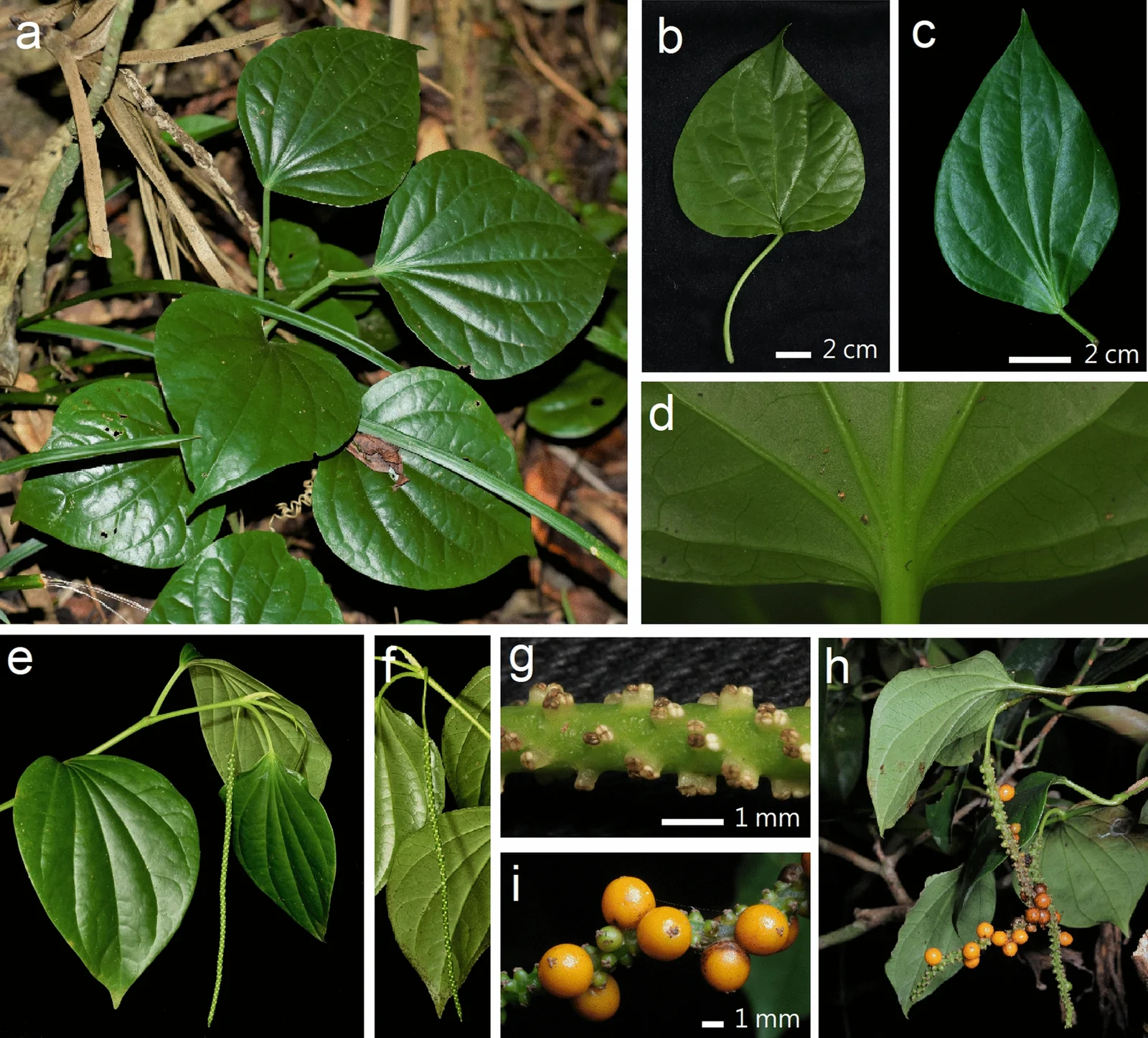
*P. interruptum* : **a** trophophyll leaves; **b** adaxial surface of trophophyll leaf; **c** adaxial surface of gonophyll leaf; **d** abaxial surface of trophophyll leaf base; **e** branch with male inflorescence; **f** male inflorescence; **g** a portion of male inflorescence;** h** branch with infructescence; **i** a portion of infructescence. Photographed by Po-Hao Chen (**a**, **b**, **d**), Kuan-Ning Kung (**c**, **g**), Wei-Yu Wang (**e**, **f**), and Tian-Chuan Hsu (**h**, **i**)

***Piper interruptum*** Opiz in C.Presl, Rel. Haenk. 1: 157 1828; Liu et al., Quart. J. Taiwan Mus. 8(4): 284. 1955; Liu and Wang in Fl. Taiwan 2: 561. 1976; Liu et al., Tre. Taiwan rev. ed. 695. 1994;, Fl. China 4: 130. 1999; Gardner, Blumea 57: 284. 2013. **Type**: Philippines: Luzon, *Haenke s.n.* (holo. PRC450583). (Figs. 6, 15a)

*Piper interruptum* var. *multinervum* **sensu** Hatusima, Mem. Fac. Agr. 7(1): 302. 1969; Lin & Lu, Fl. Taiwan 2nd ed. 2: 628. 1996; Chung and Hsu, Illustr. Fl. Taiwan. 1: 130, 2017**, non** *Piper interruptum* var. *multinervum* C.DC. (1869).

Diagnosis: *P. interruptum* is characterized by ovate to ovate-elliptic leaves with a rounded, symmetrical leaf base, venation palmate with seven primary veins, and pendulous inflorescences. It is readily distinguished from *P. betle* by having seven (vs. five) primary veins and a rounded (vs. cordate) leaf base, and from *P. kwashoense* by its thinly fleshy leaves (vs. coriaceous) and seven primary veins (vs. five).

Distribution: Taiwan: The species is found in mountainous areas at around 250 m elevation on Lanyu, where it predominantly grows in the understory.

Native Range: Taiwan, Philippines, Papuasia, Vanuatu, and Queensland (Gardner 2013).

Phenology: Flowering: May to July. Fruiting: June to September.

Conservation status: Taiwan Red List category: Data Deficient (DD) (Editorial Committee of the Red List of Taiwan Plants 2017), where this taxon was assessed under the name *P. interruptum* var. *multinervyum*. In this study, we treat it as *P. interruptum*; in Taiwan it is known from a few scattered localities on Lanyu (Taitung County).

Specimens examined: TAIWAN. Taitung: Lanyu, *P.H. Chen 3715* (TAIE); Hongtoushan, *W.L. Hsieh 1035, K.N. Kung *et al*. 2471, 2472, 2473, 2474, 2475, 2476, Kung and Hsieh 2483, K.N. Kung 2588* (CHIA), *C.E. Chang 16896* (PPI).

Notes: The phylogenetic position of *P. interruptum* is reported for the first time in the present study and is resolved within the W2 clade. This species occurs in several countries on the east side of Kano’s, Huxley’s, or Wallace’s Line and, in Taiwan, is restricted to Lanyu (see Table 1 and Taxonomic treatments), yet it is phylogenetically nested within the W2 clade (Fig. 1). This discordance between biogeographical and phylogenetic data may imply that *P. interruptum* has undergone westward range expansion, analogous to the east-to-west expansion of the widely distributed *P. macropiper* (E4 clade) (see Table 1, Taxonomic treatments, and Asmarayani 2018) but in the opposite direction. In this case, the mismatch could be attributed to insufficient knowledge of the distribution range of *P. interruptum* from western regions. Definitive resolution would require sequencing of the name-bearing type specimens.

**Fig. 7:**
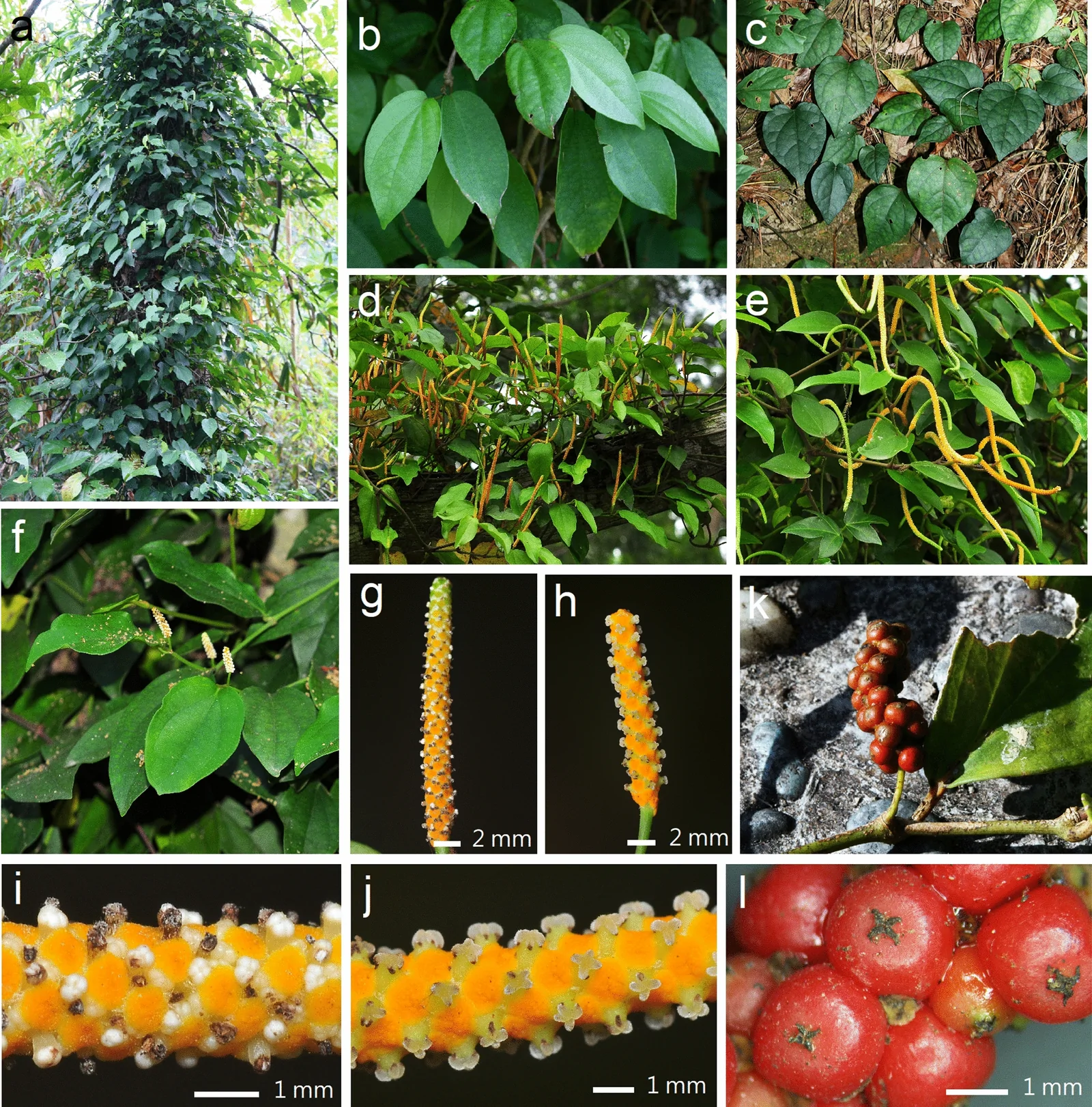
*P. kadsura* :** a** habit; **b**; gonophyll leaf** c** young leaves; **d, e** branch with male inflorescence; **f** branch with female inflorescence; **g** male inflorescence;** h** female inflorescence; **i** a portion of male inflorescence; **j** a portion of female inflorescence; **k** infructescence; **l** a portion of infructescence. Photographed by Kuan-Ning Kung (**a**–**c**, **k**, **l**), and Po-Hao Chen (**d**–**h**, **i**, **j**)

***Piper kadsura*** (Choisy) Ohwi, Acta Phytotax. Geobot. 3(2): 81. 1934. List Vasc. Pl. Taiwan 37. 1954; Liu and Wang in Fl. Taiwan 2: 408. 1976; Liu et al., Tre. Taiwan 799. 1988; Tre. Taiwan rev. ed. 695. 1994; Lin and Lu, Fl. Taiwan 2^nd^ed. 2: 628. 1996; Tseng et al., Fl. China 4: 126. 1999; Chung and Hsu, Illustr. Fl. Taiwan. 1: 131, 2017. *Ipomoea kadsura* Choisy, Mém. Soc. Phys. Genève 6:475. 1833. **Type**: Japan: “Iaponicae: Karami Kadfura, it. Saifin”. (holo. UPS-THUNB 27594). (Figs. 7, 15c, Table S5).

*Piper arboricola* C.DC., Annuaire Conserv. Jard. Bot. Genève 21: 222. 1920. **Type**: Taiwan: Ke-Lung, May 13 1903, *Faurie 480* (lecto. G00316045 designated by Gilbert and Xia 1999; isolecto. BM000611818, P00624878, P00624880 designated by Gilbert and Xia 1999).

*Piper futokadsura* Sieb., Fl. Jap. Fam. Nat. 2: 107. 1846. *nom. nud.*; Henry, List Pl. Form. 77. 1896; Hayata, J. Coll. Sci. Univ. Tokyo 25: 187. 1908; Fl. Mont. Form. 1928; Kawakami, List Pl. Form. 94. 1910; Sasaki, List Pl. Form. 141. 1928; Masamune, Short Fl. Form. 38. 1936.

*Piper hancei* Maximowicz, Bull. Acad. Imp. Sci. Saint-Pétersbourg. 31: 94. 1886; Tseng et al., Fl. China 4:128. 1999. *Chavica leptostachya* Hance, J. Bot. 6: 301. 1868, **syn. nov. Type**: China: Guangdong Province, *G.T. Sampson 13,030* (holo. K000794367).

*Piper subglaucescens* C.DC. Annuaire Conserv. Jard. Bot. Genève 21: 222. 1920. **Type**: Taiwan: Takow [Kaohsiung], Apes Hill, *Henry 715* (lecto. K000794365 here designated; isolecto. MO-204011, MO-204012, P02025610 here designated).

Diagnosis: *P. kadsura* is characterized by thinly fleshy leaves that are elliptic to elliptic**-**lanceolate, an acute leaf base, venation pinnate with two pairs of secondary veins, and erect inflorescences (occasionally recurved). It is superficially similar to *P. kawakamii*; diagnostic differences between the two species are provided under the diagnosis of *P. kawakamii*. In Taiwan, it commonly co-occurs with *P. taiwanense* but is readily distinguished by its elliptic to elliptic-lanceolate leaves (vs. ovate-lanceolate or ovate to ovate-elliptic), an acute leaf base (vs. cordate), pinnate venation (vs. palmate), and erect inflorescences (vs. pendulous).

Distribution: Taiwan: The species is widely distributed in low- to mid-elevation mountain areas across Taiwan, occurring at elevations up to 1,800 m.

Native Range: China, Japan, Korea, Taiwan, Vietnam.

Phenology: Flowering: April to August. Fruiting: June to October.

Conservation status: Taiwan Red List category: Least Concern (LC) (Editorial Committee of the Red List of Taiwan Plants 2017). In Taiwan, this species is widely distributed.

Specimens examined (representative). TAIWAN. Keelung: Keelung Islet, *T.Y.A. Yang 5578* (TNM). Taipei: Nanshijiaoshan, *P.H. Chen 1851* (PPI). Taoyuan: Shimen Reservoir, *P.H. Chen 1243* (TAIF). Taichung: Huisun Forest, *P.H. Chen 3708* (TAIE). Kaohsiung: Liguan, Southern Cross-Island Highway, *P.H. Chen 3664* (TAIE); Fenggang Forest Road, *P.H. Chen 3704* (TAIE); Lingxiangshan, *P.H. Chen 3698* (TAIE). Pingtung: Liangshan, *P.H. Chen 3714* (TAIE). Yilan: Fushan, *P.H. Chen 3716* (TAIE); Azhaowushan, *P.H. Chen 3711* (TAIE). Additional specimens examined are listed in Data S6.

Notes: Given that our phylogenies indicate *P. arboricola*, *P. hancei*, *P. kadsura*, and *P. subglaucescens* represent a single species (Figs. 1, 2), we critically reviewed their morphological descriptions in the literature and examined types along with additional herbarium specimens (Table S5). Based on our assessments and in accordance with the ICN (Turland et al. 2025), we confirm that *P. kadsura* is the correct name for this historically important herbal medicine (Kim et al. 2011; Yang et al. 2021). Moreover, the placement of *P. kadsura* in the W2 clade and *P. wallichii* within the W3 clade (Fig. 1) indicates that they are different species, thereby refuting the hypothesis proposed by Tseng et al.’s (1999) that *P. kadsura* may represent a form of *P. wallichii*.

De Candolle (1920) mentioned Henry, A., 715 as the type of *P. subglaucescens* which were collected from the Apes hill [Shoushan], Formosa [Taiwan] and kept in the Herbarium Museum Paris (P), Royal Botanic Gardens, Kew (K), and Missouri Botanical Garden (MO). There are four specimen copies of A. Henry, 715 [K000794365, MO-204011, MO-204012, P02025610], all of which were equally well preserved. Among them, two are male specimens (K000794365, MO-204012) and two are female (MO-204011, P02025610). We then designated K000794365 as the lectotype here, with MO-204011, MO-204012, and P02025610 as isolectotypes.

**Fig. 8:**
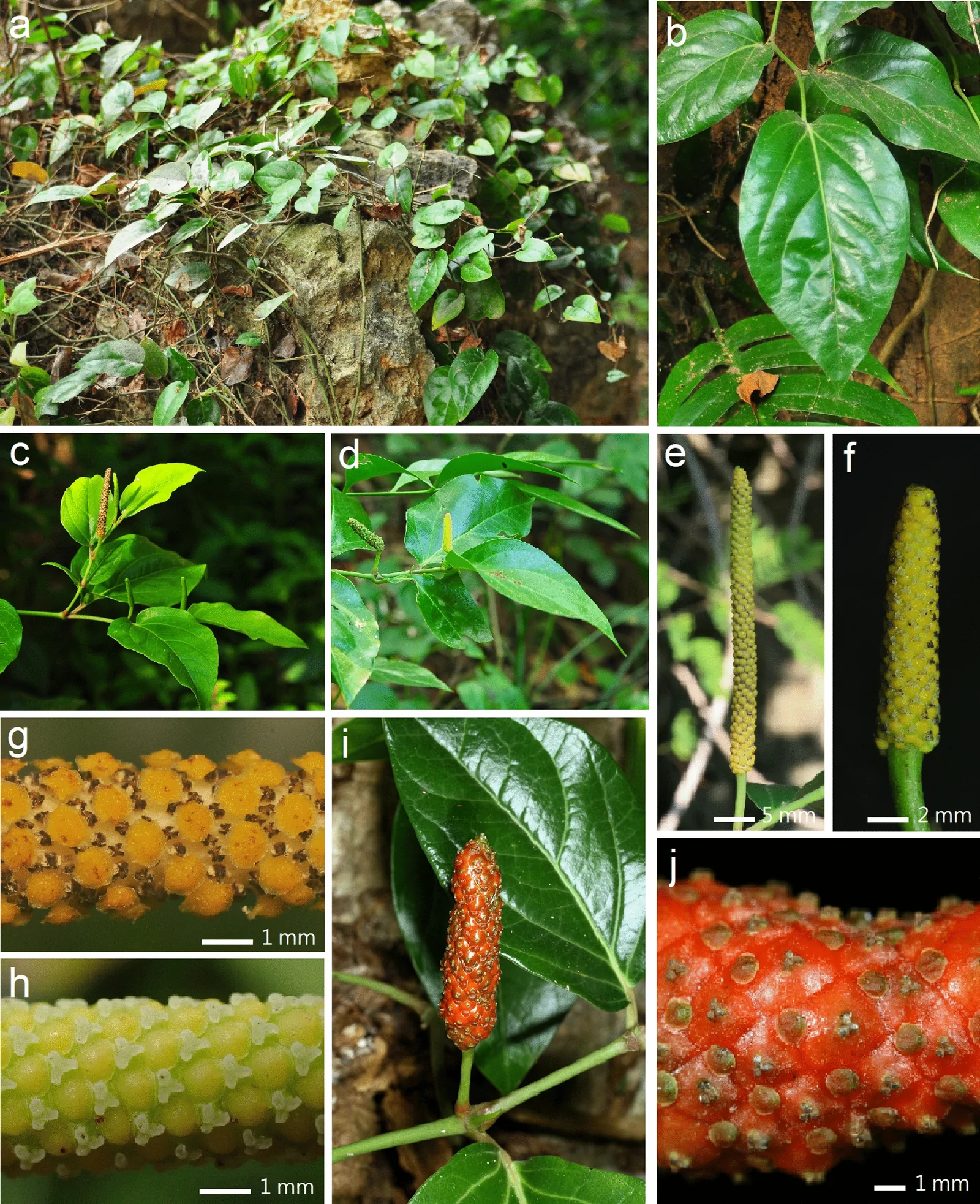
*P. kawakamii* : **a** habit; **b** gonophyll leaf; **c** branch with male inflorescence; **d** branch with female inflorescence; **e** male inflorescence; **f** female inflorescence; **g** a portion of male inflorescence;** h** a portion of female inflorescence; **i** infructescence; **j** a portion of infructescence. Photographed by Po-Hao Chen (**a**–**d**, **f**–**h**), and Kuan-Ning Kung (**e**, **i**, **j**)

***Piper kawakamii*** Hayata, J. Coll. Sci. Imp. Univ. Tokyo 30(1): 234. 1911. Sasaki, List Pl. Form. 141. 1928; Masamune, Short Fl. Form. 38. 1936; List Vasc. Pl. Taiwan 37. 1954; Liu et al., Quart. J. Taiwan Mus. 8(4): 285. 1955; Liu and Wang in Fl. Taiwan 2: 562. 1976; Liu et al., Tre. Taiwan 799. 1988; Tre. Taiwan rev. ed. 696. 1994; Lin and Lu, Fl. Taiwan 2nd ed. 2: 629. 1996; Tseng et al., Fl. China 4: 127. 1999; Chung and Hsu, Illustr. Fl. Taiwan. 1: 131, 2017. **Type**: Taiwan: Koshun: Kurarusha, Juli. 2, 1906, *T. Kawakami 1645* (iso. TAIF 7082). (Figs. 8, 15a).

*Piper subcordata* Hayata, Gen. Ind. Fl. Form. 61. 1917. nom. nud. Kawakami, List Pl. Form. 94. 1910; Sasaki, List Pl. Form. 141. 1928; Masamune, Short Fl. Form. 38. 1936; List Vasc. Pl. Taiwan 37. 1954; Liu et al., Quart. J. Taiwan Mus. 8(4): 284. 1955.

Diagnosis: *P. kawakamii* is characterized by elliptic to elliptic-lanceolate or ovate to ovate-elliptic leaves, an often asymmetrical leaf base, venation pinnate with four pairs of secondary veins, and erect inflorescences. It is readily distinguished from the morphologically similar *P. kadsura* by its papery leaves and four (vs. two) pairs of secondary veins, and further by having an acute to asymmetrical leaf base, whereas *P. kadsura* typically has thinly fleshy leaves and a leaf base that is usually rounded to cordate.

Distribution: Taiwan: The species is distributed in uplifted coral reef areas below 400 m in elevation in Kaohsiung and Pingtung.

Native Range: Endemic to Taiwan.

Phenology: Flowering: April to August. Fruiting: May to September.

Conservation status: Taiwan Red List category: Least Concern (LC) (Editorial Committee of the Red List of Taiwan Plants 2017). In Taiwan, this species is restricted to uplifted coral reefs in Kaohsiung and Pingtung.

Specimens examined (representative). TAIWAN. Kaohsiung: Shoushan, *P.H. Chen 3709* (TAIE); Shoushan, *P.H. Chen 3710* (TAIE); Beishoushan, *K.N. Kung 850* (CHIA); Beishoushan, *W.P. Leu 959* (HAST); Chaishan, behind Longquan Temple, *Y.H. Tseng 2364* (TCF); Fengshan, *C.F. Chen 1454* (PPI). Pingtung: Sheding, *Kung and Ku 208* (CHIA); Kenting National Park, *T.G. Lammers 8478* (HAST); Eluanbi, *T.C. Huang 7862* (TAI); Xiaoliuqiu, M.Y. Shen 4748 (TAIE). Additional specimens examined are listed in Data S6.

Notes: This study is the first to report the phylogenetic position of *P. kawakamii*, which is resolved within the E5 clade.

**Fig. 9:**
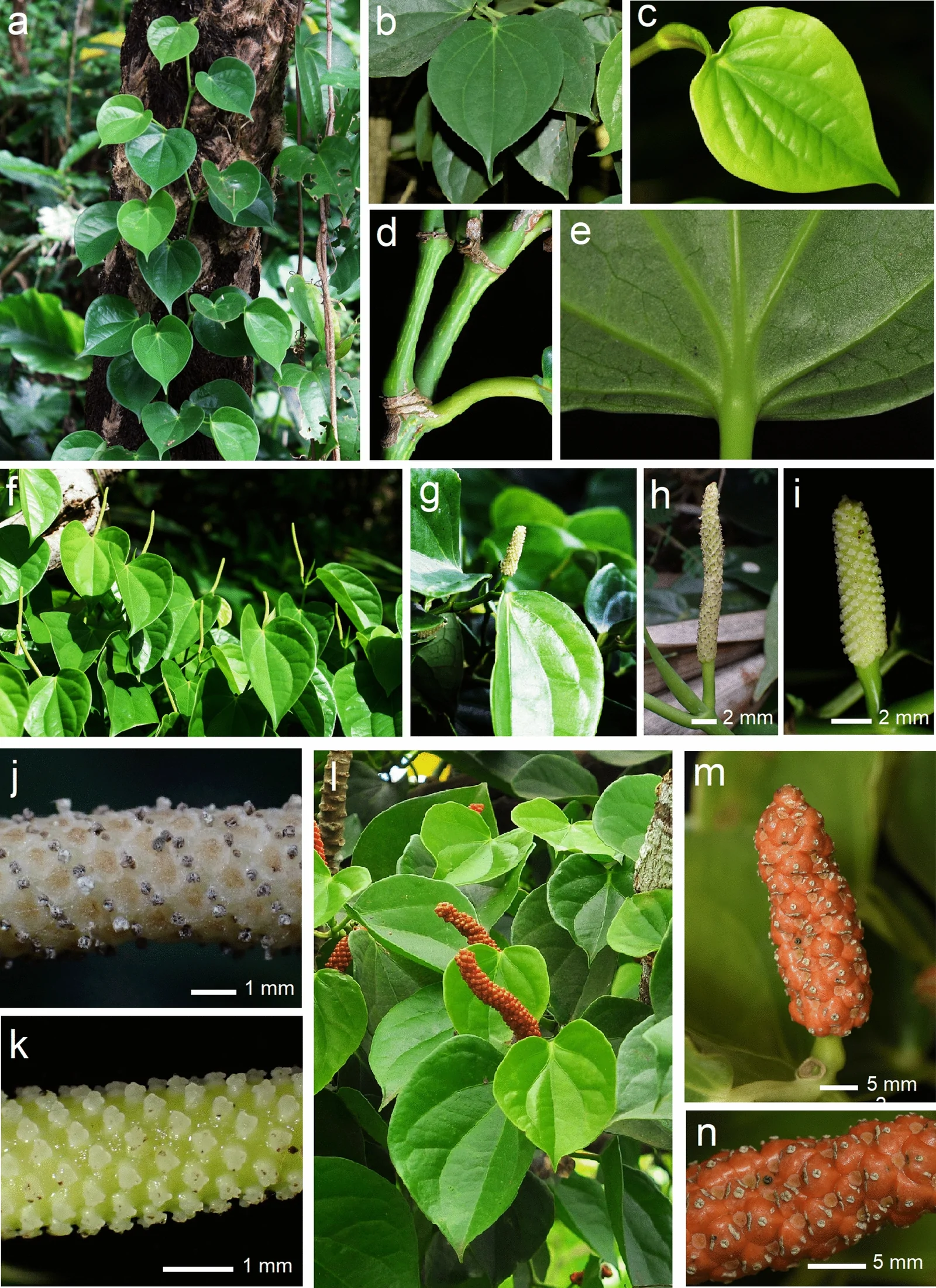
*P. kwashoense* : **a** habit; **b** gonophyll leaf; **c** young leaves; **d** branch; **e** abaxial surface of trophophyll leaf base; **f** branch with male inflorescence; **g** branch with female inflorescence;** h** male inflorescence; **i** female inflorescence; **j** a portion of male inflorescence; **k** a portion of male inflorescence; **l** branch with infructescence; **m** infructescence; **n** a portion of infructescence. Photographed by Kuan-Ning Kung (**a**, **c**, **d**, **f**, **g**, **l**–**n**), and Po-Hao Chen (**b**, **e**, **h**–**k**)

***Piper kwashoense*** Hayata, J. Coll. Sci. Univ. Tokyo 30 (1): 235. 1911; Liu et al., Quart. J. Taiwan Mus. 8(4): 285. 1955; Liu and Wang in Fl. Taiwan 2: 564. 1976; Liu et al., Tre. Taiwan rev. ed. 696. 1994; Tseng et al., Fl. China 4: 127. 1999. **Type**: Taiwan: Kwashoto [Lyudao], Aug. 15, 1907, *Kawakami and Kobayashi 4751* (holo. TAI115533). (Fig. 9, 15d, Table S5).

*Piper lanyuense* K.N.Kung and Kun C.Chang, Ann. Bot. Fennici 57: 94. 2020, **syn. nov. Type**: Taiwan: Taitung: Lanyu Township, Tatienchih, 22°00′59.1″ N, 121°33′47.36″ E, ca. 180 m a.s.l., July 20, 2009, *K.N. Kung *et al*. 1118* (♀ holo. CHIA; iso. TAIF).

*Piper insectifugum* **sensu** Gardner, Blumea 57: 284. 2013, **non** *Piper insectifugum* C.DC. ex Seem.

*Piper philippinum* **sensu** Liu et al., Quart. J. Taiwan Mus. 8(4): 285. 1955; Hatusima, Mem. Fac. Agr. 7(1): 302. 1969; Liu and Wang in Fl. Taiwan 2: 564. 1976; Liu et al., Tre. Taiwan rev. ed. 696. 1994; Lin and Lu, Fl. Taiwan 2nd ed. 2: 629. 1996; Gardner, Blumea 51: 583. 2006; Chung and Hsu, Illustr. Fl. Taiwan. 1: 132, 2017, **non** *Piper philippinum* Miq. (1844).

Diagnosis: *P. kwashoense* is characterized by coriaceous leaves, leaves cordate or ovate to ovate-elliptic, venation palmate with five primary veins, and erect inflorescences. It is readily distinguished from the sympatric climbers *P. betle* and *P. interruptum* by its erect (vs. pendulous) inflorescences and coriaceous (vs. thinly fleshy) leaves. In addition, leaf-base morphology in *P. kwashoense* is variable (cordate, rounded, or acute), whereas *P. betle* typically has a cordate leaf base and *P. interruptum* a rounded leaf base.

Distribution: Taiwan: The species is found on Lanyu and Ludao, typically occurring in the understory or along forest edges at elevations below 400 m.

Native Range: Endemic to Taiwan.

Phenology: Flowering: May to August. Fruiting: June to September.

Conservation status: Taiwan Red List category: Least Concern (LC) (Editorial Committee of the Red List of Taiwan Plants 2017). In Taiwan, this species is restricted to Ludao and Lanyu (Taitung County).

Specimens examined (representative). TAIWAN. Taitung: Lanyu, *P.H. Chen 3727* (TAIE); Datianchi, *P.H. Chen 2218* (PPI); Lanyu, *C.E. Chang 5563* (PPI); Lanyu Township, *S.Y. Lu 17480* (TAIF); Central Highway–Yeyin, *K.N. Kung *et al*. 613* (CHIA); Xiaotianshi, *T.Y.A. Yang *et al*. 3419* (TNM); Hongtoushan, *C.I Peng 6619* (HAST); Yeyin Yongxing Farm, *W.P. Leu 2153* (HAST). Ludao: *C.E. Chang 12340* (PPI); *T.T. Chen 9077* (TAIF). Additional specimens examined are listed in Data S6.

Notes: Our thorough investigation of type specimens and additional materials, as well as our comparative analysis of morphological traits from the literature (Table S5), suggests recognizing *P. kwashoense, P. insectifugum*, and *P. philippinum* as distinct. Based on our morphological examination, *P. lanyuense* should therefore be treated as a synonym of *P. kwashoense*. In Taiwan, *P. kwashoense* typically has glabrous petioles and leaves and a coriaceous lamina, whereas *P. insectifugum* has been described as having a very finely pubescent petiole and a membranous lamina. *P. kwashoense* also usually differs from *P. philippinum* by its erect inflorescences (vs. often flexible or pendulous) and subglobose fruits (vs. ovoid fruits). These characters help clarify past confusion among the three names in the Taiwanese literature.

A notable clarification concerns the type specimen of *P. kwashoense*, which was collected by Kawakami and Kobayashi 475 (TAI) from Ludao in 1907 (Hayata 1911; Hsu et al. 2023), rather than from Lanyu in 1899 by K. Miyake s.n. (TI), as previously misinterpreted by Chang and Kung (2020). Hsu et al. (2023), referencing Z. Kobayashi’s collection records, clarified that the specimen number ‘*475*’ was incorrectly cited and should be corrected to ‘*4751*’.

Moreover, in the present study, the phylogenetic position of *P. kwashoense* is reported for the first time and is placed within the E5 clade (Fig. 1). Our ITS tree places both *P. kwashoense* and *P. insectifugum* within the E5 clade but reveals that they have independent evolutionary trajectories (Fig. S2). Specifically, *P. kwashoense* forms a clade with *P. urdanetanum* C.DC., a species occurring in the Philippines (POWO 2025), while *P. insectifugum* is sister to *P. austrocaledonicum* C.DC., which is distributed from New Guinea to the Solomon Islands (POWO 2025). Although *P. philippinum* was not sampled in our molecular analysis and could not be evaluated in that context, its distinct morphological traits support its recognition as a separate species. Taken together, our findings confirm that *P. kwashoense* is endemic to Taiwan, with its distribution restricted to Lanyu and Ludao.

**Fig. 10:**
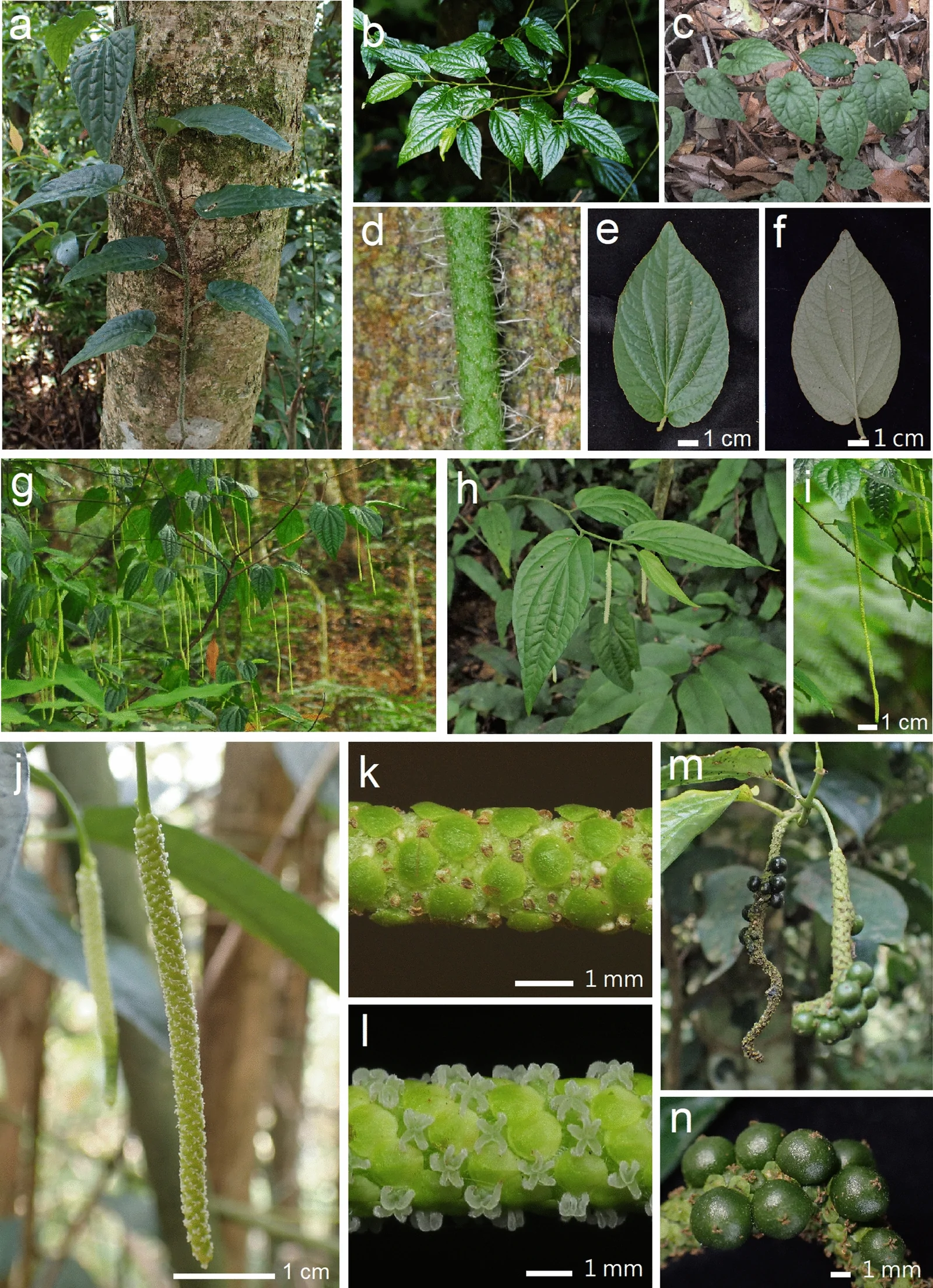
*P. laosanum* : **a** habit; **b** gonophyll leaves; **c** trophophyll leaves; **d** branch; **e** adaxial surface of gonophyll leaf; **f** abaxial surface of gonophyll leaf; **g** branch with male inflorescence;** h** branch with female inflorescence; **i** male inflorescence; **j** female inflorescence; **k** a portion of male inflorescence; **l** a portion of female inflorescence; **m** infructescences; **n** a portion of infructescence. Photographed by Po-Hao Chen (**a**, **c**–**n**), and Kuan-Ning Kung (**b**)

***Piper laosanum*** C.DC., Annuaire Conserv. Jard. Bot. Genève 2: 269–270. 1898. **Type**: Laos: systemate fluviali Attopeu, *F.J. Harmand 1364* (lecto. P02025519; designated by Suwanphakdee et al. 2023; isolecto. P02025520, P02025521). (Fig. 10, 15d, Table S5).

*Piper sintenense* Hatusima, Acta Phytotax. Geobot. 4: 210. 1935; Masamune, List Vasc. Pl. Taiwan 37. 1954; Lin and Lu, Fl. Taiwan 2^nd^ed. 2: 629. 1996; Tseng et al., Fl. China 4: 122. 1999; Chung and Hsu, Illustr. Fl. Taiwan. 1: 133, 2017, **syn. nov. Type**: Taiwan: Taipei, Kanko[乾溝], Nov. 1932, *S. Hatusima s.n.* (iso. TAI034290).

*Piper arboricola* **sensu** Liu and Wang in Fl. Taiwan 2: 561. 1976; Liu et al., Tre. Taiwan 798. 1988; Tre. Taiwan rev. ed. 696. 1994, **non** *Piper arboricola* C.DC.

*Piper hispidum* Hayata, J. Coll. Sci. Imp. Univ. Tokyo 30: 234. 1911; Sasaki, List Pl. Form. 141. 1928; Masamune, Short Fl. Form. 38. 1936; List Vasc. Pl. Taiwan 37. 1954, **nom. illeg**. **non** *Piper hispidum* Kunth (1816). **Type**: Taiwan: Koshun [Pingtung 屏東], Garanbi [Oluanpi], 1896, *Y. Tashiro s.n.* (holo. TI-T00298).

Diagnosis: *P. laosanum* is characterized by papery leaves that are ovate**-**lanceolate or ovate to ovate**-**elliptic, a usually asymmetrical leaf base, venation pinnate with three pairs of secondary veins, and pendulous inflorescences. Notably, the upper leaf surface is distinctly hispid or pilose, a conspicuous character that readily separates *P. laosanum* from the other Taiwanese *Piper* species treated here.

Distribution: Taiwan: The species is widely distributed across low- to mid- elevation mountainous regions, primarily inhabiting forest understories and humid, semi-shaded environments.

Native Range: Laos, Thailand (Suwanphakdee et al. 2023), and Taiwan.

Phenology: Flowering: April to July. Fruiting: June to October.

Conservation status: Taiwan Red List category: Least Concern (LC) (Editorial Committee of the Red List of Taiwan Plants 2017), where this taxon was assessed under the name *P. sintenense*. It is widely distributed in Taiwan.

Specimens examined (representative). TAIWAN. Keelung: Nuannuan, *C.C. Wang *et al*. 61* (TNM). Taipei: Shizitoushan, *P.H. Chen 1839* (PPI). Miaoli: Xiangtianhushan, *P.H. Chen 3852* (TAIF). Nantou: Lugu, *P.H. Chen 695* (PPI); Houjianshan, *P.H. Chen 3399* (TAIE). Kaohsiung: Fenggang Forest Road, *P.H. Chen 3705* (TAIE); Wangzishan–Minghaishan, *P.H. Chen 3913* (TAIF). Pingtung: Laisheshan, *P.H. Chen 2248* (PPI). Taitung: Daren, *P.H. Chen 2021* (PPI). Yilan: Fushan, *S.W. Chung 2297* (TAIF). Additional specimens examined are listed in Data S6.

Notes: *P. sintenense* was formerly regarded as endemic to Taiwan (excluding Lanyu and Ludao) (Hatusima 1935; Lin and Lu 1996; Tseng et al. 1999), but it may be conspecific with either *P. laosanum* or *P. hongkongense* C.DC. (Tseng et al. 1999). *P. hongkongense* was not included in our sampling, so it is not discussed further. Our phylogeny shows that the *P. laosanum* sample nests within *P. sintenense* samples in the W1 clade (Fig. S2), supporting their conspecificity. Based on detailed examination of types and additional specimens, along with a thorough comparison of morphological characteristics from their descriptions (Table S5), we confirm that *P. laosanum* and *P. sintenense* represent the same species. Following the principle of priority in the International Code of Nomenclature for algae, fungi, and plants (ICN) (Turland et al. 2025), *P. laosanum* is recognized as the correct name for this taxon. This species is not endemic to Taiwan. Its disjunct distribution across Laos, Thailand, and Taiwan might suggest a history of long-distance dispersal (Metschina et al. 2025; Sen et al. 2019; Valentin-Silva et al. 2021) or insufficient sampling in adjacent regions.

**Fig. 11:**
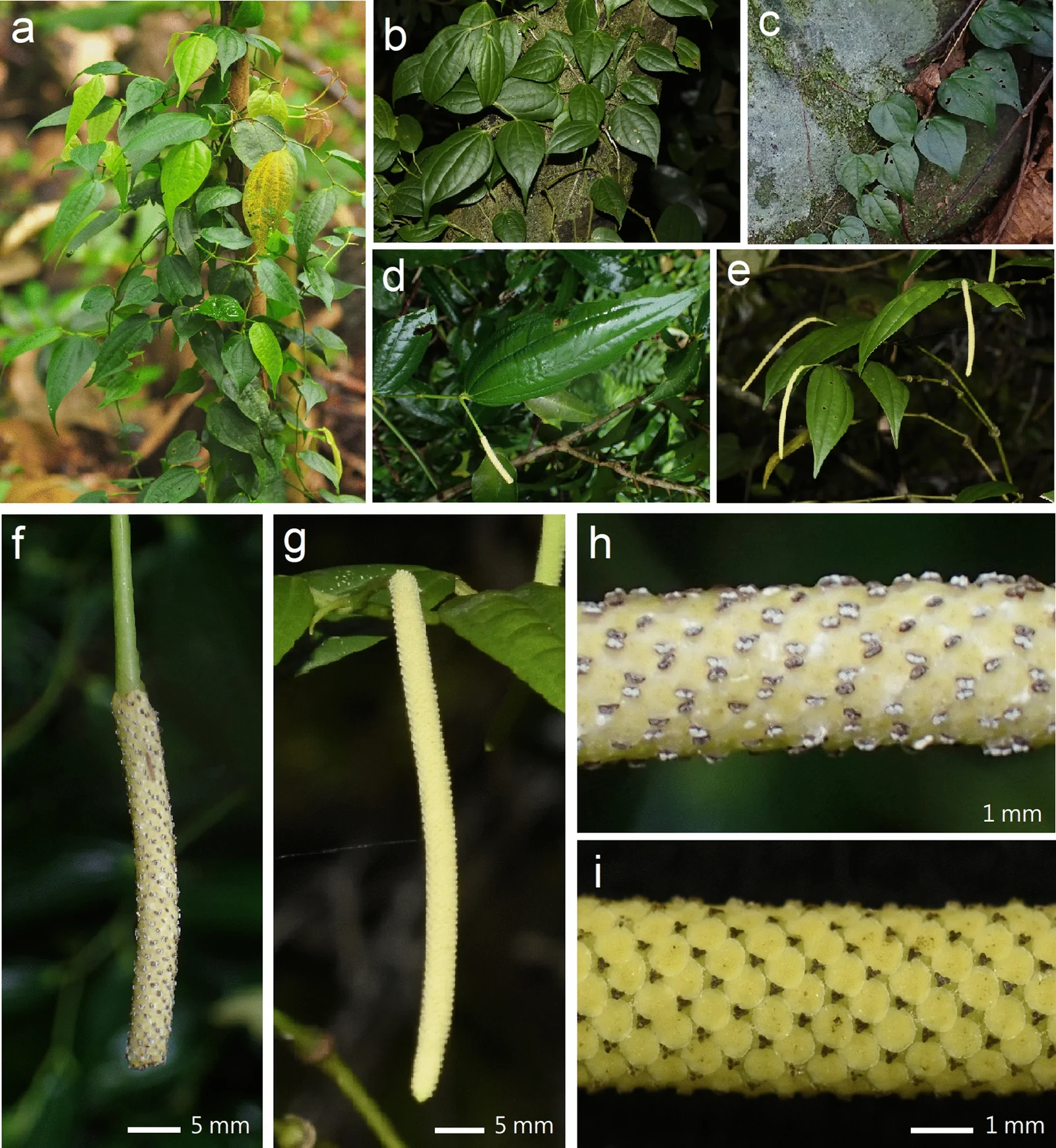
*P. macropiper* : **a** habit; **b** gonophyll leaves; **c** young leaves; **d** branch with male inflorescence; **e** branch with female inflorescence; **f** male inflorescence; **g** female inflorescence;** h** a portion of male inflorescence; **i** a portion of female inflorescence. All photographs by Po-Hao Chen

***Piper macropiper*** Pennant, Outl. Globe 4: 242. 1800; Gardner, Blumea 51: 582. 2006. *Piper arborescens* Roxb., Hort. Bengal.: 80. 1814; Liu and Wang in Fl. Taiwan 2: 560. 1976; Liu et al., Tre. Taiwan 798. 1988; Tre. Taiwan rev. ed. 695. 1994; Lin and Lu, Fl. Taiwan 2nd ed. 2: 628. 1996; Tseng et al., Fl. China 4: 124. 1999; Gardner, Blumea 57: 287. 2013; Chung and Hsu, Illustr. Fl. Taiwan. 1: 129, 2017. **Type**: Rumphius, Herb. Amboin. 5: t. 28, f.1 1747 (lecto. designated by Turner 2013). (Figs. 11, 5e, Table S5).

*Piper arborescens* Roxb. var. *angustilimbum* Quis., Philip. J. Sci. 43: 22. 1930; Liu et al., Quart. J. Taiwan Mus. 8(4): 284. 1955; Hatusima, Mem. Fac. Agr. 7(1): 302. 1969. **Type**: Philippines: Luzon, Tayabas Prov., Lucban, May 1907, *A.D.E. Elmer 7384* (holo. PNH (destroyed); iso. GH00005942, NY002839F16).

*Piper kotoense* Yamam., J. Soc. Trop. Agr. 4: 304. 1932; Masamune, List Vasc. Pl. Taiwan 37. 1954. **Type**: Taiwan: Taitung: Lanyu, Orchid Is., June 15, 1911, *S. Sasaki s.n.* (syn. TAI034277).

Diagnosis: *P. macropiper* is characterized by papery leaves that are typically ovate-lanceolate, glabrous, with venation palmate with five primary veins, and pendulous inflorescences. It is readily distinguished from other Lanyu-sympatric climbers (e.g., *P. betle*, *P. interruptum*, and *P. kwashoense*) by its papery leaf texture and ovate-lanceolate leaf shape.

Distribution: Taiwan: The species is found on Lanyu Island, where it primarily grows in the forest understory in dark, humid environments.

Native Range: India, Taiwan, Malaysia, Indonesia, Philippines, Vanuatu, Micronesia, Australia, Wallis, Futuna Is., and Samoa (Gardner 2013; POWO 2025; Suwanphakdee et al. 2020).

Phenology: Flowering: May to August. Fruiting: July to September.

Conservation status: Taiwan Red List category: Least Concern (LC) (Editorial Committee of the Red List of Taiwan Plants 2017), where this taxon was assessed under the name *P. arborescens*. It is known only from Lanyu (Taitung County) in Taiwan.

Specimens examined (representative). TAIWAN. Taitung: Lanyu, *P.H. Chen 3728* (TAIE); Hongtoushan, *P.H. Chen 2197* (TAIE); Lanyu, *K.N. Kung *et al*. 667* (CHIA); Lanyu, *S.Y. Lu 17478* (TAIF); Lanyu, *C.E. Chang 16659 *(PPI); Tianchi, *K.N. Kung *et al*. 2597* (CHIA); Tianchi, *T.C. Huang *et al*. 9372* (TAI); Tianchi, *Wang and Lai 6229* (NTUF); Hongtoushan, *C.E. Chang 16804* (PPI); Qingsheshan, *Kung and Hsieh 2564* (CHIA). Additional specimens examined are listed in Data S6.

Notes: *P. arborescens* has been widely recognized in earlier studies, and both *P. arborescens* var. *angustilimbum* and *P. kotoense* are considered synonyms of this species (Chang and Kung 2020; Chung and Hsu 2017; Editorial Committee of the Red List of Taiwan Plants 2017; Lin and Lu 1996; Tseng et al. 1999). However, *P. arborescens* is regarded as a synonym of *P. macropiper* (Merrill 1948; Turner 2013), a species whose occurrence in Taiwan has been suggested in Gardner’s work (2006, 2010, 2013). Our phylogenetic analysis reveals that the *P. macropiper* sample is nested within the *P. arborescens* samples in clade E4 (Figs. 1, S2), with strong branch supports (pp = 1.00, bs = 97), suggesting that they represent the same species. By integrating our molecular evidence with a thorough investigation of type specimens and additional herbarium materials, along with a comprehensive analysis of morphological descriptions from the literature (Table S5), we conclude that *P. arborescens* is conspecific with *P. macropiper*, and that the presence of *P. macropiper* in Taiwan is confirmed.

**Fig. 12:**
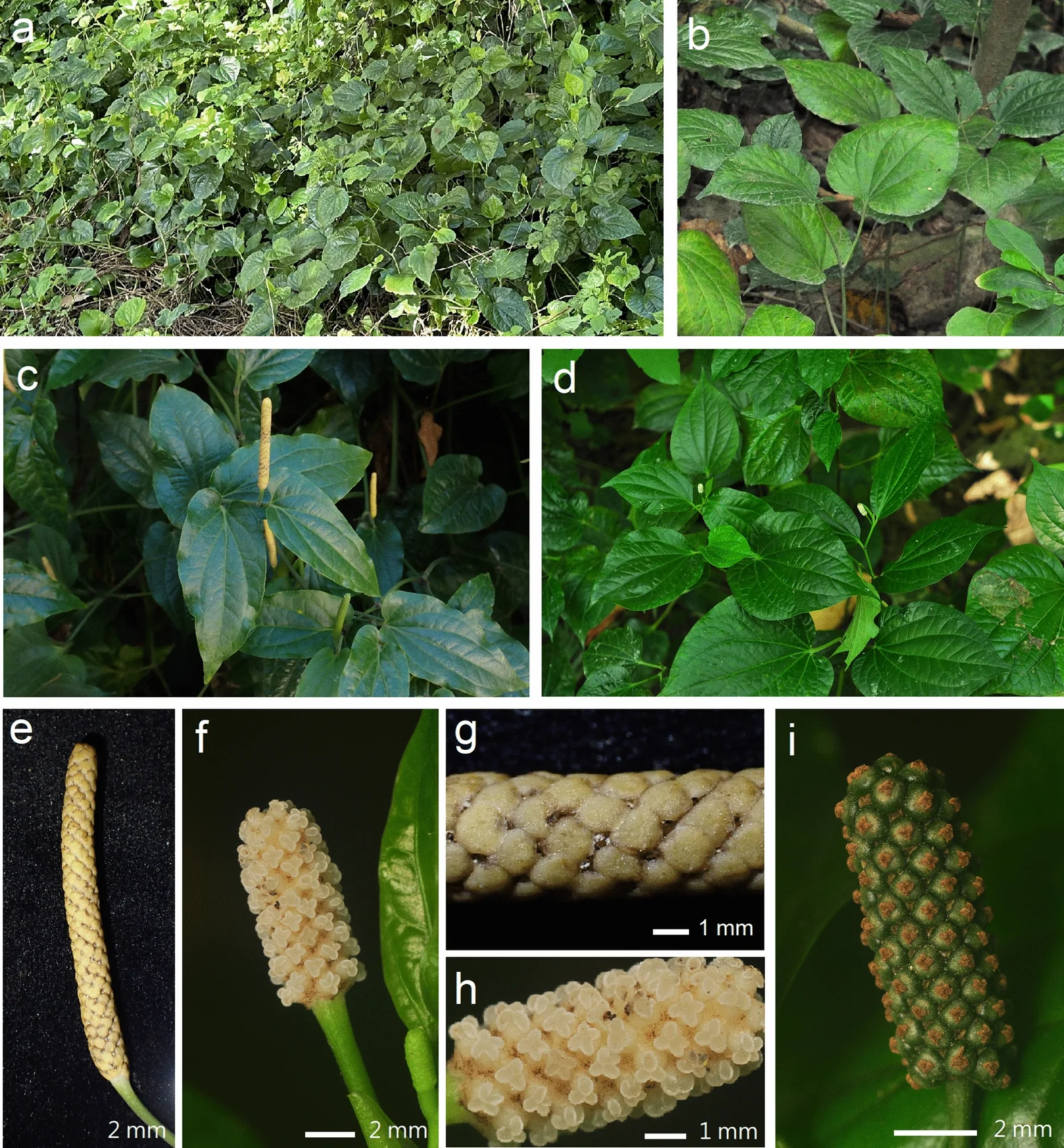
*P. sarmentosum* : **a** habit; **b** gonophyll leaves; **c** branch with male inflorescence; **d** branch with female inflorescence; **e** male inflorescence; **f** female inflorescence; **g** a portion of male inflorescence;** h** a portion of female inflorescence; **i** infructescence. All photographs by Po-Hao Chen

***Piper sarmentosum*** Roxb. Asiat. Res. 11: 565. 1810; Chung and Hsu, Quart. J. Forest. Res. 38(2): 2, 2016; Chung and Hsu, Illustr. Fl. Taiwan. 1: 132, 2017. **Type**: “cultivated in Calcutta”, Roxburgh tab. 1267 (lecto. K designated by Gilbert and Xia 1999). (Figs. 12, 15b).

Diagnosis: *P. sarmentosum* is characterized by its prostrate, herbaceous habit, papery, cordate leaves**,** glabrous foliage, solitary spikes, and unisexual flowers**.** The prostrate, herbaceous growth form readily separates it from the other Taiwanese *Piper* species treated here, which are predominantly lianas or trees. It is further distinguished from *P. umbellatum* by leaves with length and width approximately equal (vs. broader than long), glabrous leaf surfaces (vs. puberulent)**,** single inflorescences (vs. umbellate), and unisexual flowers (vs. bisexual).

Distribution: Taiwan: Naturalized. The species is distributed along low-elevation forest margins of southern Taiwan.

Native Range: India, Sri Lanka, Myanmar, China, Laos, Vietnam, Cambodia, Malaysia, Indonesia, and Philippines (POWO 2025; Suwanphakdee et al. 2020).

Phenology: Flowering and fruiting: April to November.

Conservation status: Taiwan Red List category: Not Applicable (NA) (Editorial Committee of the Red List of Taiwan Plants 2017). *P. sarmentosum* is treated as a naturalized species in Taiwan and is therefore listed as NA in the Red List.

Specimens examined: TAIWAN. Taoyuan: Jiulutoushan, *T.C. Hsu 6255* (TAIF), *T.C. Hsu 6725* (TAIF). Hsinchu: Sixth Cemetery, *T.C. Hsu 12303* (TAIF). Nantou: Zhongliao, *K.W. Li 169* (PPI). Chiayi: Jenitan Reservoir, *Z.H. Chen 1619* (TAIF). Kaohsiung: Panpingshan, *S.W. Chung and T.H. Chen 12336* (TAIF); *P.H. Chen 1329* (TAIF); Gushan, *P.H. Chen 3090* (TAIE).

Notes: *P. sarmentosum* is a naturalized species in Taiwan and is readily distinguishable from other Taiwanese *Piper*.

**Fig. 13:**
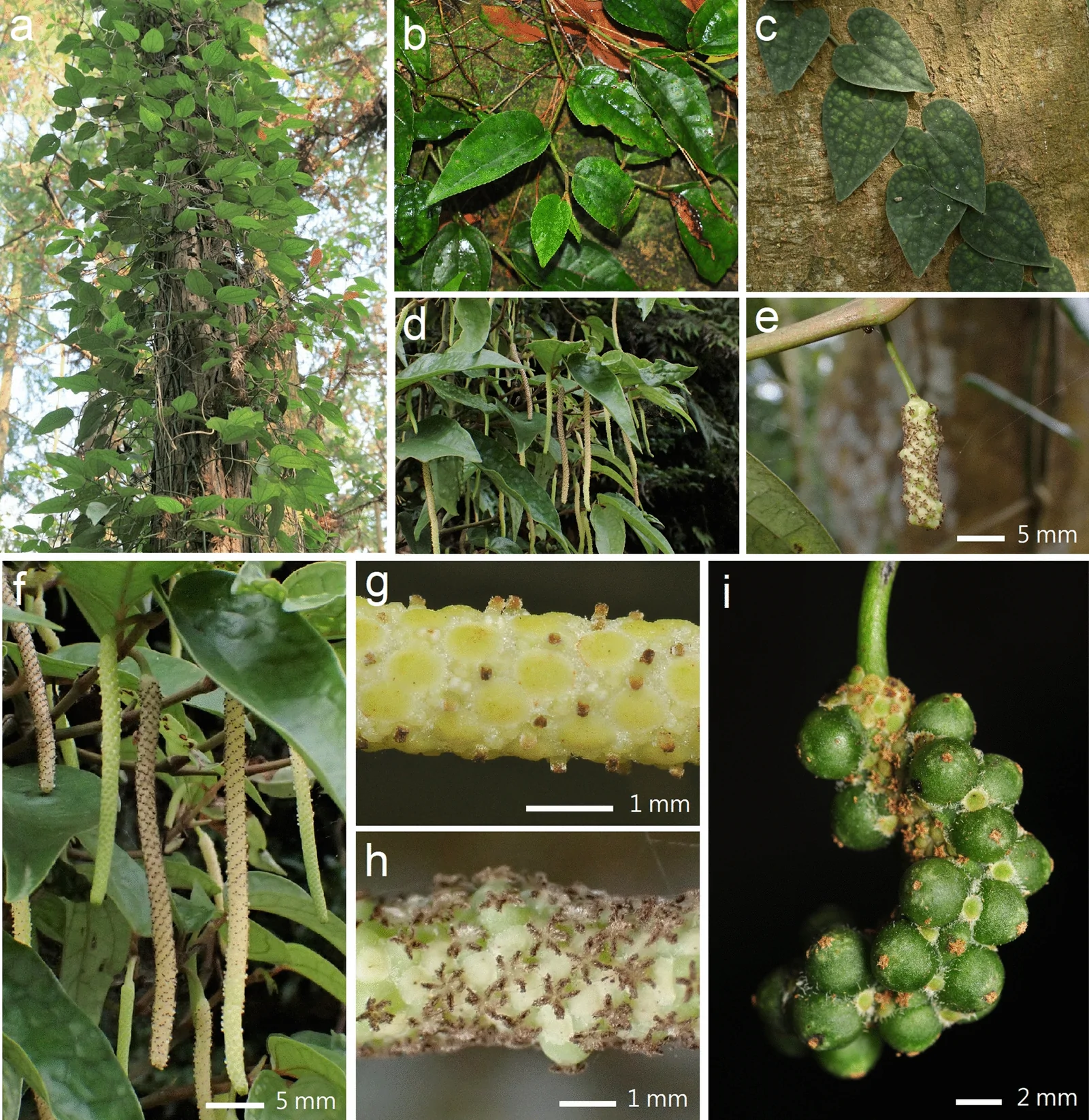
*P. taiwanense* : **a** habit; **b** gonophyll leaves; **c** young leaves; **d** branch with male inflorescence; **e** female inflorescence; **f** male inflorescence; **g** a portion of male inflorescence;** h** a portion of female inflorescence; **i** infructescence. Photographed by Kuan-Ning Kung (**a**), and Po-Hao Chen (**b**–**i**)

***Piper taiwanense*** Lin and Lu, Taiwania 40(4): 356. *f*.4. 1995: Lin and Lu, Fl. Taiwan 2nd ed. 2: 629. Pl. 302. 1996; Tseng et al., Fl. China 4: 122. 1999; Chung and Hsu, Illustr. Fl. Taiwan. 1: 133, 2017. **Type**: Taiwan: Taichung, Tungshih [東勢], May 20, 1995, *S.Y. Lu 24,728* (holo. TAIF 177777; iso. TAIF). (Figs. 13, 15e).

Diagnosis: *P. taiwanense* is characterized by ovate-lanceolate or ovate to ovate-elliptic leaves, a cordate leaf base, venation palmate with three primary veins, and pendulous inflorescences. It is most similar to *P. betle* but can be distinguished by a generally smaller leaf blade (< 10 cm long and < 5 cm wide vs. usually > 10 cm long and > 5 cm wide), a shorter petiole (< 1.5 cm vs. > 1.5 cm), and venation palmate with three primary veins (vs. five primary veins). In Taiwan, *P. taiwanense* commonly co-occurs with *P. kadsura*; diagnostic differences between these two species are provided under the diagnosis of *P. kadsura*.

Distribution: The species is distributed across low- to mid-elevation mountain areas in Taiwan, primarily occurring below 1,200 m, in forest margins or shaded habitats within forests.

Native Range: Endemic to Taiwan.

Phenology: Flowering: April to September. Fruiting: June to October.

Conservation status: Taiwan Red List category: Least Concern (LC) (Editorial Committee of the Red List of Taiwan Plants 2017). In Taiwan, this species is widely distributed.

Specimens examined (representative). TAIWAN. Taipei: Zhishanyan, *Nonaka and Mori s.n.* (TAI). Taichung: Dongshi, *Lin and Lu 24728* (Holo.) (TAIF). Nantou: Lugu, *P.H. Chen 694* (PPI). Kaohsiung: Fenggang Forest Road, *P.H. Chen 3699* (TAIE); Lingxiangshan, *P.H. Chen 3706* (TAIE). Pingtung: Manzhou Township, Nanrenshan, *Y.C. Lu *et al*. 1497* (HAST); Xiaoliuqiu, *T. Hosokawa 1668* (TAI); Pengjishan, *P.H. Chen 396* (PPI). Taitung: Dahu Township, Tushan, *K.N. Kung *et al*. 834* (CHIA). Hualien: Wanrong Township, Hongye Village, *C.I Peng *et al*. 11603* (HAST). Additional specimens examined are listed in Data S6.

Notes: This study is the first to determine the phylogenetic position of *P. taiwanense*, placing it within the W3 clade.

**Fig. 14:**
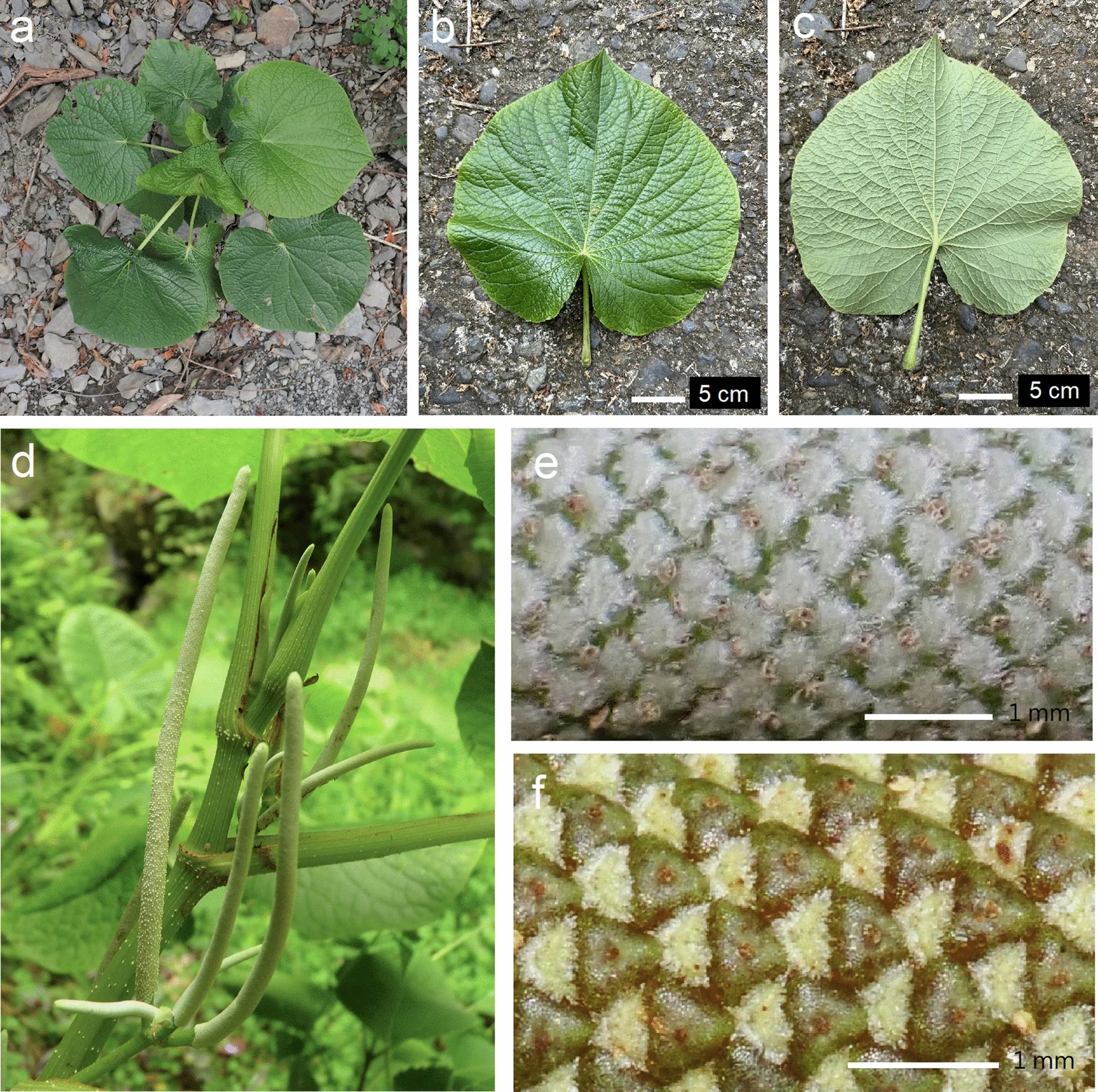
*P. umbellatum* :** a** habit; **b** adaxial surface of gonophyll leaf; **c** abaxial surface of gonophyll leaf; **d** inflorescence; **e** a portion of inflorescence; **f** a portion of infructescence. All photographs by Po-Hao Chen

**Fig. 15:**
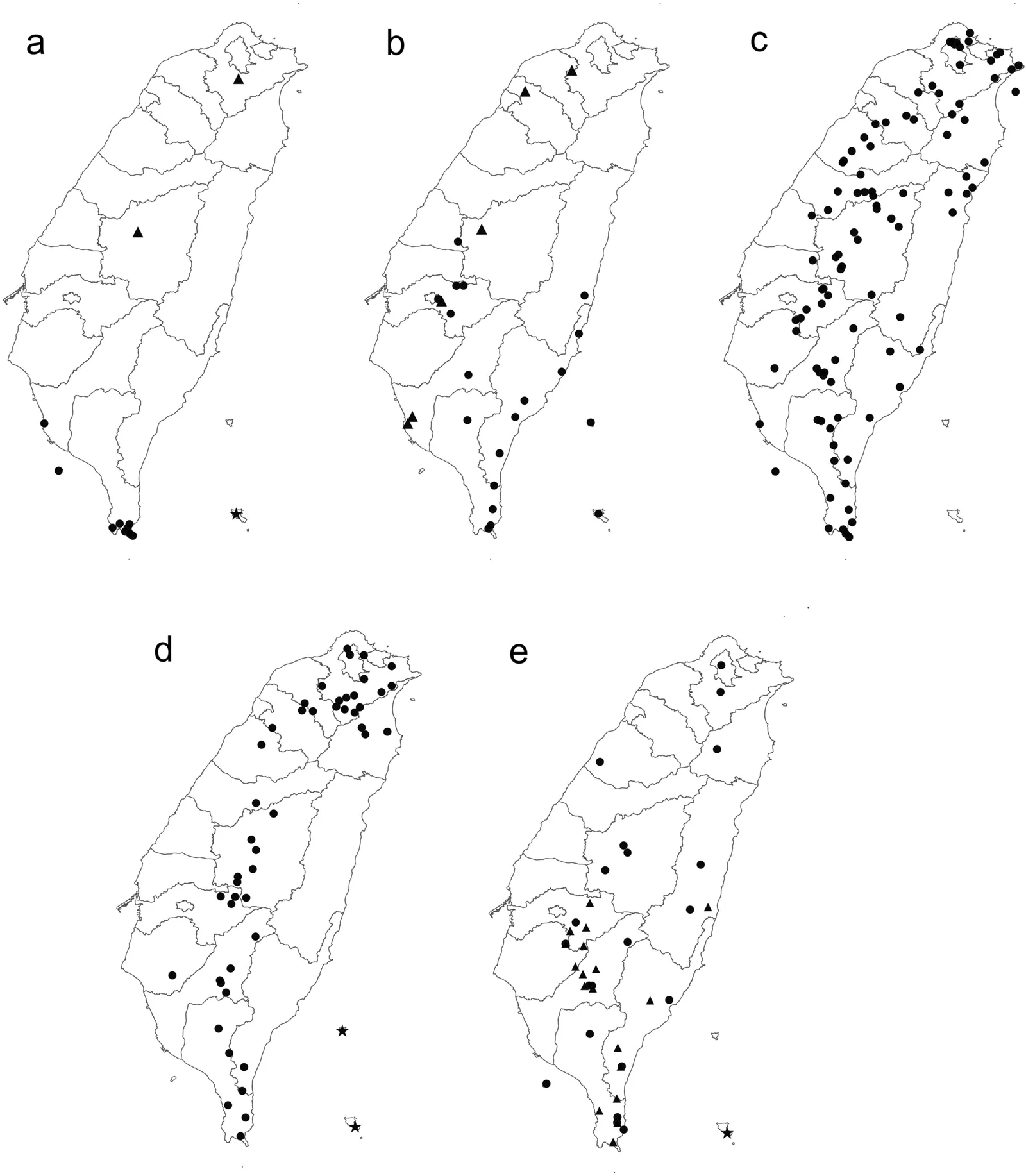
Distribution maps of Taiwanese *Piper* species. **a** *Piper aduncum* (▲), *P. kawakamii* (●), and *P. interruptum* (★); **b** *P. betle* (●) and *P. sarmentosum* (▲); **c** *P. kadsura* (●); **d** *P. laosanum* (●) and *P. kwashoense* (★); **e** *P. macropiper* (★), *P. taiwanense* (●), and *P. umbellatum* (▲)

***Piper umbellatum*** L., Sp. Pl. 30. 1753; Willd., Sp. Pl. **1**:167. 1797; Nov. Gen. Sp. (quarto ed.) **1**:59. 1815; C.DC., Philip. J. Sci. Bot. **5**:463. 1910; Liu et al., Quart. J. Taiwan Mus. **4**:285. 1955 Liu and Wang in Fl. Taiwan **2**:564. 1976; Makino and Nemoto, Fl. Jap. 158. 1931; Lin and Lu, Fl. Taiwan 2nd ed. **2**:631. 1996; Tseng et al., Fl. China 4:129. 1999. **Type**: Plumier, Descr. Pl. Amér., 53, t. 73. 1693 (lecto. designated by Huber 1987). (Figs. 14, 15e).

*Piper subpeltatum* Willd., Sp. Pl. **1**:166. 1797; Henry, List Pl. Form. 77. 1896; Sasaki, List Pl. Form. 141. 1928; Masamune, Short Fl. Form. 38. 1936; List Vasc. Pl. Taiwan 37. 1954; Kawakami, List Pl. Form. 94. 1910; Wang, Taxon. Rev. Ord. Pip. Taiwan 34. 1973; Yang, List Pl. Taiwan 476. 1982; Liu et al., Tre. Taiwan 799. 1988; Tre. Taiwan rev. ed. 696. 1994. **Type**: Amboina, Lomba; Rumphius, Herb. Amb. 6: 133, t. 59/1. 1750.

Diagnosis: *P. umbellatum* is characterized by its small shrubby habit, large, cordate leaves with a long petiole (> 10 cm), venation palmate with 7–9 primary veins, and erect, umbellate inflorescences with bisexual flowers. The combination of a shrubby growth form, very long petioles, and umbellate inflorescences readily distinguishes *P. umbellatum* from the other Taiwanese *Piper* species treated here. A detailed comparison with the morphologically similar *P. sarmentosum* is provided under the diagnosis of *P. sarmentosum*.

Distribution: Taiwan: Naturalized. The species is primarily distributed in the southern and eastern mountainous regions of Taiwan, typically occurring in humid or shaded habitats at elevations below 600 m.

Native Range: Neotropical areas (POWO 2025; Roersch 2010; Smith et al. 2008).

Phenology: Flowering: April to July. Fruiting: June to September.

Conservation status: Taiwan Red List category: Least Concern (LC) (Editorial Committee of the Red List of Taiwan Plants 2017). In the present study, *P. umbellatum* is considered a naturalized species in Taiwan; accordingly, we treat it here as Not Applicable (NA) in the context of Taiwan, and recommend updating the Red List assessment under the revised status.

Specimens examined (representative). TAIWAN. Chiayi: Dapu Township, Bats Cave, *C.H. Chen 518* (HAST). Tainan: Dongshan Township, Kantoushan Trail, *K.N. Kung *et al*. 65* (CHIA). Kaohsiung: Shanping, *J.C. Wang *et al*. 9467* (HAST); Weiliaoshan, *P.H. Chen 2464* (PPI); *P.H. Chen 3809* (TAIE). Pingtung: Manzhou Township, Nanrenshan, *C.E. Chang 20427* (PPI); Nanren Lake, *H.J. Su 9587* (NTUF); *S.H. Liu 2133* (TAIF). Taitung: Biru, *P.F. Lu 3272* (TAIF). Hualien: Qimei–Ruisui, *Y. Yamamoto 1361* (TAI). Additional specimens examined are listed in Data S6.

Notes: *P. umbellatum* is a naturalized species in Taiwan and is readily distinguishable from other Taiwanese *Piper*.

## Discussions

### Additional evolutionary insights into Taiwanese *Piper* based on phylogeny

In addition to supporting the taxonomic revisions proposed above, the morphological similarity observed between *P. kadsura* and *P. wallichii* (Tseng et al. 1999), which are phylogenetically distant (Fig. 1), highlights the need for additional research on trait evolution and convergence within Asian *Piper* (Asmarayani 2018; Losos 2011; Valentin-Silva et al. 2021).

Moreover, our phylogenetic results (Figs. 1, S2) differ from those of earlier studies that inferred *P. kadsura* to be closely related to *P. polysyphonum* C.DC. (Asmarayani 2018) and *P. leptostachyum* Wall. ex Miq. (= *P. chaudocanum* C.DC.) (Sen et al. 2019). We carefully reviewed the relevant literature and examined the types of *P. polysyphonum* and *P. leptostachyum* (Suwanphakdee et al. 2016, 2020; Tseng et al. 1999) and asserted that these two taxa can be readily distinguished morphologically from *P. kadsura*. The incongruence between our and Asmarayani (2018) analyses on *P. polysyphonum* is likely attributable to the insufficient sampling in both studies within the W2 clade (Fleming et al. 2023; Som 2015; Steenwyk et al. 2023). In our phylogenetic analysis (Fig. S2), the *P. leptostachyum* sample (= *P. chaudocanum*; GenBank accession # EU581152) from Sen (2019) does not cluster with the *P. leptostachyum* sample (GenBank accession # EU581275) reported by Jaramillo et al. (2008) but instead falls within the *P. kadsura* complex, suggesting that it was likely misidentified and should be assigned to *P. kadsura*.

### Bidirectional spread of Taiwanese *Piper* species across biogeographical regions

Incorporating molecular phylogenetic and biogeographical data (Fig. 1 and Table 1), our results suggest that three of the seven native Taiwanese species––*P. kwashoense* (E5 clade), *P. macropiper* (E4 clade), and *P. taiwanense* (W3 clade)––have remained on their respective side of origin, lending support to both Kano’s line and the phytogeographical island concept (Kano 1933, 1935a, b; Li and Keng 1950). In contrast, three other native species exhibit varying degrees of range expansion across the boundary (Fig. 1 and Table 1). *P. kawakamii* (E5 clade) occurs in the Hengchun Peninsula and has expanded northwestward into parts of southern Taiwan. *P. kadsura* (W2 clade) and *P. laosanum* (W1 clade) are distributed throughout the rest of Taiwan, with the former expanding southeast into the Hengchun Peninsula and Ludao, and the latter also expanding southeast into the Hengchun Peninsula. The distribution patterns, together with the overlap of the WWL and EWL clades in the Hengchun Peninsula and Ludao, point to a highly complex evolutionary history of *Piper* in Taiwan, likely shaped by recurrent dispersal events across biogeographical boundaries.

To the best of our knowledge, this is the first study to demonstrate, using both biogeographical data and molecular evidence, the bidirectional expanding and evolutionary processes of a single genus across the northernmost extent of Huxley’s Line, thereby extending earlier research that relied solely on unidirectional dispersal patterns in different genera based on biogeographical data (e.g., Hsu and Wolf 2009; Huang 2011; Kanehira 1935; Kano 1933, 1935a, b; Keng 1956; Li and Keng 1950; Liu and Ling 1978; Liu and Liu 1977; Masamune 1939).

## Conclusion

In summary, our morphological and molecular analyses revise the taxonomy of the genus *Piper* in Taiwan and clarify the evolutionary relationships of Taiwanese species and their close relatives. We recognize 11 species in Taiwan, including four naturalized and three endemic species. Among the seven native Taiwanese species, four belong to the WWL clades and three to the EWL clades, with the phylogenetic placements of *P. interruptum*, *P. kawakamii*, *P. kwashoense*, and *P. taiwanense* reported here for the first time. Our data underpin the taxonomic revisions proposed here for *P. sintenense*, *P. arborescens*, and *P. kadsura*, and confirm the name of *P. kwashoense*. In addition, when combined with biogeographical evidence, our findings provide the first documented case—and an ideal study system—for exploring bidirectional expansions with phylogenetic implications in a genus occurring at the northernmost extent of Huxley’s Line.

Here, we also propose key directions for future phylogenetic and phylogeographical studies on Taiwanese *Piper*. Continued studies—involving denser taxon sampling within clades occurring in Taiwan, coupled with population-level analyses (e.g., Kuo et al. 2024; Liu et al. 2023; Tsai et al. 2015)—are needed to clarify the relationships among Taiwanese *Piper* and its closely related taxa in the clades as well as to elucidate the bidirectional expansion history of Taiwanese *Piper*. Particular attention might also be given to the mechanisms underlying the origination of endemic species, the chronology of colonization events, and the timing and frequency of boundary crossings.

## Supplementary Information

Below is the link to the electronic supplementary material.

Supplementary file1 (PDF 993 kb)

Supplementary file2 (TXT 3 kb): Data S1. Morphological character scores and codes.

Supplementary file3 (TXT 136 kb): Data S2. Alignment and tree files of the trnL-F region.

Supplementary file4 (TXT 137 kb): Data S3. Alignment and tree files of the ITS region.

Supplementary file5 (TXT 249 kb): Data S4. Concatenated alignment and tree files.

Supplementary file6 (TXT 74 kb): Data S5. Alignment and tree files based on the ITS region for the Piper kadsura complex.

Supplementary file7 (DOCX 25 kb): Data S6. The list of additional specimens examined in the present study.

## Data Availability

The GenBank accession numbers for all sequences analyzed in this study are provided in Table 1 and Fig. 3. Newly generated sequences will be made publicly available upon publication.
