# Supplementary material for: Phylogeny and taxonomic revisions of Piper (Piperaceae) in Taiwan
Source: J Plant Res. 2026 Aug 5;139(5):685–716. doi: 10.1007/s10265-026-01733-0 (PMC13588929; doi:10.1007/s10265-026-01733-0)
Supplement: Supplementary file 1 [file 10265_2026_1733_MOESM1_ESM.pdf]

Phylogeny and taxonomic revisions of *Piper* (Piperaceae) in Taiwan

Journal of Plant Research

Po-Hao Chen [1,2], Chia-Hao Chang [3], Kuan-Ning Kung [4], Hung-Chih Lin [5], An-Ching Chung [1,2], Chia-Ying Ou [6], Shih-Hui Liu [6,*]

[1] Graduate Institute of Bioresources, National Pingtung University of Science and Technology, 1 Shuefu Rd., Neipu Township, Pingtung County 91201, Taiwan

[2] Lioukuei Research Center, Taiwan Forest Research Institute, 198 Zhongzhuang, Chunghsing Village, Lioukuei Dist., Kaohsiung City 844005, Taiwan

[3] Science Education, National Taipei University of Education, Taipei City 106320, Taiwan

[4] Chiayi Research Center, Taiwan Forest Research Institute, Ln. 432, Wenhua Rd., West Dist., Chiayi City 600054, Taiwan

[5] Division of Silviculture, Taiwan Forest Research Institute, 53, Nanhai Rd., 100051 Taipei, Taiwan

[6] Biological Sciences, National Sun Yat-sen University, Kaohsiung City 804201, Taiwan

***Corresponding author:** Biological Sciences, National Sun Yat-sen University, Kaohsiung City 804201, Taiwan. E-mail: liushihhui@gmail.com. ORCID: 0000-0002-5429-6869.

Table S1. The samples included in this study, along with their scientific names and GenBank accession numbers, are provided. For newly collected samples, additional details such as sample numbers, collectors, collection numbers, localities, and the herbaria where they are deposited are also included. Taxa marked with asterisks (*), number signs (#), and dollar signs ($) represent synonyms of *Piper laosanum*, *P. macropiper*, and *P. leptostachyum* respectively.

**Taxon**	**Sample number**	**ITS**	***trn*L-F**	**Collector *collection* #**	**Locality**	**Herbarium**
**Outgroups**						
Houttuynia cordata Thunb.		EU329272	AF332969			
Manekia naranjoana (C.DC.) Callejas ex N. Zamora, Hammel & Grayum		HQ731559	EF422821			
Manekia sydowii (Trel.) T. Arias, Callejas & Bornst.		HQ731554	EF422820			
Peperomia pellucida (L.) Kunth		MH050267				
Peperomia serpens (Sw.) Loudon		FJ424446				
**Ingroups**						
Piper abbreviatum Opiz		MH493092	MH476599			
Piper acre Blume		MH493102	MH476603			
Piper arborescens Roxb. #	15	PQ893330	PQ892194	P.H. Chen *2197*	Taiwan: Taitung County	PPI

Piper arborescens Roxb. #	27	PQ893335	PQ892206	P.H. Chen *3728*	Taiwan: Taitung County	TAIE
Piper arcuatum C. Presl		MH493109	MH476606			
Piper arfakianum C.DC.		MH493113	MH476607			
Piper argyrites Ridl. ex C.DC.		MH493122	MH476608			
Piper argyrophyllum Miq.		DQ868704				
Piper attenuatum Buch.-Ham. ex Miq.		MH128790				
Piper austrocaledonicum C.DC.		MH493124	MH476609			
Piper baccatum Blume		OM338975	OM414727			
Piper bantamense Blume		MH493151	MH476657			
Piper bavinum C.DC.		AF275199				
Piper betle L.	17	PQ893321	PQ892196	P.H. Chen *3712*	Taiwan: Pingtung County	TAIE
Piper betle L.	18	PQ893322	PQ892197	P.H. Chen *2005*	Taiwan: Taitung County	PPI
Piper betle L.	19	PQ893323	PQ892198	P.H. Chen *3713*	Taiwan: Pingtung County	TAIE
Piper betle L.	25	PQ893333	PQ892204	P.H. Chen *3726*	Taiwan: Taitung County	TAIE
Piper betle L.	32	PQ893340	PQ892211	C.Y. Ou *2301*	Taiwan: Taitung County	TAIF
Piper betle L.		EF060065	MH476624			
Piper blumei (Miq.) Backer		MH493212	MH476672			
Piper boehmeriifolium (Miq.) Wall. ex C.DC.		KF924140				
Piper bojonyum C.DC.		MH493219	MH476623			

Piper borneense N.E. Br.	MH493229	MH476626
Piper breviamentum C.DC.	EU581134	
Piper brevifolium C.DC.	MH493232	MH476716
Piper buruanum Miq.	MH493235	MH476627
Piper caducibracteum C.DC.	MH493239	MH476638
Piper caninum Blume	EF060067	MH476633
Piper capense L. f.	AY326200	
Piper celebicum Blume	MH493279	MH476636
Piper celtidiforme Opiz	MH493283	MH476709
Piper chaudocanum C. DC. $	EU581152	
Piper chlorostachyum C.DC.	MH493286	MH476715
Piper cilibracteum C.DC.	MH493306	MH476643
Piper cubeba L. f.	EF060070	MH476645
Piper curtisii C.DC.	MH493321	MH476646
Piper cyrtostachys Ridl.	MH493327	MH476647
Piper decumanum L.	AF275203	MH476648
Piper dumiformans C.DC.	MH493338	MH476723
Piper excelsum G. Forst.	DQ868716	
Piper febrifugum C.DC.	MH493344	MH476649
Piper flaviflorum C.DC.	MH493352	MH476650

Piper flavimarginatum C.DC.		MH493347	MH476735			
Piper fragile Benth.		MH493355	MH476654			
Piper fragrans Trel.		MH493381	MH476717			
Piper galeatum (Miq.) C.DC.		MH128775				
Piper gibbilimbum C.DC.		MH493666	MH476596			
Piper griffithii C.DC.		EU581225				
Piper guahamense C.DC.		MZ308629				
Piper guineense Schumach. & Thonn.		MH493384	MH476656			
Piper hancei Maxim.		MZ602145	EU519602			
Piper hancei Maxim.		MZ636730				
Piper hancei Maxim.	28	PQ893336	PQ892207	Y.H. Cheng *2709*	China: Hong Kong	TNM
Piper hapnium Buch.-Ham.		MH128773				
Piper hooglandii (I. Hutton & P.S. Green) M.A. Jaram.		AF275192				
Piper hookeri Miq.		MH128793				
Piper hymenophyllum Miq.		AY572327				
Piper insectifugum C.DC.		MZ308634				
Piper interruptum Opiz	23	PQ893326	PQ892202	P.H. Chen *3715*	Taiwan: Taitung County	TAIE
Piper kadsura (Choisy) Ohwi	4	PQ893311	PQ892183	P.H. Chen *3698*	Taiwan: Kaohsiung City	TAIE

Piper kadsura (Choisy) Ohwi	5	PQ893312	PQ892184	P.H. Chen *3704*	Taiwan: Kaohsiung City	TAIE
Piper kadsura (Choisy) Ohwi	6	PQ893313	PQ892185	P.H. Chen *3664*	Taiwan: Kaohsiung City	TAIE
Piper kadsura (Choisy) Ohwi	8	PQ893329	PQ892187	J.T. Chang *s.n.*	Japan: Kagoshima Prefecture	TAIE
Piper kadsura (Choisy) Ohwi	9	PQ893315	PQ892188	J.T. Chang *s.n.*	Japan: Kagoshima Prefecture	TAIE
Piper kadsura (Choisy) Ohwi	10	PQ893316	PQ892189	J.T. Chang *s.n.*	Japan: Kagoshima Prefecture	TAIE
Piper kadsura (Choisy) Ohwi	11	PQ893317	PQ892190	P.H. Chen *3708*	Taiwan: Nantou County	TAIE
Piper kadsura (Choisy) Ohwi	14	PQ893320	PQ892193	P.H. Chen *3711*	Taiwan: Taichung City	TAIE
Piper kadsura (Choisy) Ohwi	20	PQ893324	PQ892199	P.H. Chen *3714*	Taiwan: Pingtung County	TAIE
Piper kadsura (Choisy) Ohwi	24	PQ893327	PQ892203	P.H. Chen *3716*	Taiwan: Ilan County	TAIE
Piper kadsura (Choisy) Ohwi	29	PQ893337	PQ892208	T.C. Hsu *15077*	Japan: Okinawa Prefecture	TAIF
Piper kadsura (Choisy) Ohwi	30	PQ893338	PQ892209	T.C. Hsu *15078*	Japan: Okinawa Prefecture	TAIF
Piper kadsura (Choisy) Ohwi	33	PQ893341	PQ892212	S.H. Liu *2180*	Taiwan: Miaoli County	TAIF
Piper kadsura (Choisy) Ohwi	35	PQ893343	PQ892214	M.T. Hsieh *s.n.*	Taiwan: Keelung City	TAIE
Piper kadsura (Choisy) Ohwi	36	PQ893344	PQ892215	M.T. Hsieh *s.n.*	Taiwan: Keelung City	TAIE
Piper kadsura (Choisy) Ohwi	37	PQ893345	PQ892216	C.Y. Ou *2404*	Taiwan: Kaohsiung City	TAIF
Piper kadsura (Choisy) Ohwi	40	PQ893346	PQ892217	C.Y. Ou *2407*	Taiwan: Kaohsiung City	TAIF
Piper kadsura (Choisy) Ohwi		EF450290	MH476658			

Piper kawakamii Hayata	12	PQ893318	PQ892191	P.H. Chen *3709*	Taiwan: Kaohsiung City	TAIE
Piper kawakamii Hayata	13	PQ893319	PQ892192	P.H. Chen *3710*	Taiwan: Kaohsiung City	TAIE
Piper kurzii Ridl.		MH493399	MH476659			
Piper kwashoense Hayata	21	PQ893325	PQ892200	P.H. Chen *2218*	Taiwan: Taitung County	PPI
Piper kwashoense Hayata	26	PQ893334	PQ892205	P.H. Chen *3727*	Taiwan: Taitung County	TAIE
Piper laosanum C.DC.		AY572326				
Piper leptostachyum Wall. ex Miq.		EU581275				
Piper lessertianum C.DC.		MH493408	MH476662			
Piper lolot C.DC.		AY326208				
Piper longivaginans C.DC.		EU581282				
Piper macropiper Pennant		MH493412	MH476664			
Piper majusculum Blume		MH493444	MH476670			
Piper medinillifolium Quisumb.		AY667455				
Piper melchior (Sykes) M.A. Jaram.		AF275191				
Piper mestonii F.M. Bailey		MH493447	MH476673			
Piper methysticum G. Forst.		MH493667	MH476595			
Piper mollissimum Blume		MH493454	MH476676			
Piper montivagum Ridl.		MH493670	MH476597			
Piper mullesua Buch.-Ham. ex D. Don		MH109310	AY032651			
Piper muricatum Blume		EF060076	MH476722			

Piper myrmecophilum C.DC.		AY572328				
Piper nigrum L.	7	PQ893314	PQ892186	P.H. Chen *3707*	Taiwan: Kaohsiung City	TAIE
Piper nigrum L.		EF450283	LC637549			
Piper nigrum L.		MZ636719				
Piper novoguineense Warb.		MH493489	MH476713			
Piper oreophilum Ridl.		MH493496	MH476706			
Piper pedicellatum C.DC.		MG208073				
Piper pedicellosum Wall.		MH493501	MH476682			
Piper pentandrum C.DC.		MH493505	MH476731			
Piper philodendron Ridl.		MH493509	MH476683			
Piper pierrei C.DC.		AF275200				
Piper polysyphonum C.DC.		MG208065				
Piper porphyrophyllum N.E. Br.		MH493513	MH476684			
Piper pseudonigrum Velayudhan & Amalraj		MH128782				
Piper puberulirameum C.DC.		MH493526	MH476687			
Piper pullibaccum Trel.		MH493528	MH476660			
Piper quinqueangulatum Miq.		MH493531	MH476690			
Piper ramipilum C.DC.		MH493559	MH476694			

Piper relictum Lekhak, Kambale & S.R.Yadav		MH128783				
Piper retrofractum Vahl		AF275196	MH476695			
Piper retrofractum Vahl		EF060081				
Piper rhytidocarpum Hook. f.		MH128806				
Piper ribesioides Wall.		MH128803	MH476697			
Piper rostratum Roxb.		MH493577	MH476704			
Piper rubro-venosum Hort ex Rodigas		MH493582	MH476698			
Piper sarcopodum C.DC.		MH493587	MH476732			
Piper sarmentosum Roxb.	22	PQ893332	PQ892201	P.H. Chen *3090*	Taiwan: Kaohsiung City	PPI
Piper sarmentosum Roxb.		KF924096	MH476702			
Piper schizonephos C.DC.		MH493598	MH476734			
Piper sintenense Hatus. *	2	PQ893328	PQ892181	P.H. Chen *3705*	Taiwan: Kaohsiung City	TAIE
Piper sintenense Hatus. *	16	PQ893331	PQ892195	P.H. Chen *2021*	Taiwan: Taitung County	PPI
Piper subcaniramum C.DC.		MH493602	MH476707			
Piper sulcatum Blume		MH493607	MH476708			
Piper taiwanense Lin & Lu	1	PQ893309	PQ892180	P.H. Chen *3699*	Taiwan: Kaohsiung City	TAIE
Piper taiwanense Lin & Lu	3	PQ893310	PQ892182	P.H. Chen *3706*	Taiwan: Kaohsiung City	TAIE
Piper thomsonii (C.DC.) Hook. f.		MG208068				
Piper trichostachyon (Miq.) C.DC.		MH128780				

Piper truncatibaccum C.DC.		MH493611	MH476710			
Piper umbellatum L.	31	PQ893339	PQ892210	P.H. Chen *3809*	Taiwan: Kaohsiung City	TAIE
Piper umbellatum L.	34	PQ893342	PQ892213	S.H. Liu *2133*	Taiwan: Pingtung County	TAIF
Piper umbellatum L.		EF060088	MW925711			
Piper uncinulatum Ridl.		MH493617	MH476711			
Piper urdanetanum C.DC.		AF275207				
Piper versteegii C.DC.		MH493619	MH476712			
Piper wallichii (Miq.) Hand.-Mazz.		EF450289	LC637552			
Piper wightii Miq.		MH128796	KJ635844.1			

Table S2. The universal primers, optimal annealing temperatures, and product sizes for the ITS and *trn*L-F regions in this study.

DNA regions	Primers	Primer Sequences (5' -->3')	Primer Sources	Annealing Temperature (°C)	Product Size (bp)
**Nuclear region**					
ITS (ITS1 + 5.8S + ITS2)	ITS_4	TCCTCCGCTTATTGATATGC	White et al. 1990	57	692-721
	ITS_5	GGAAGTAAAAGTCGTAACAAGG	White et al. 1990		
**Chloroplast regions**					
*trn*L-F (tRNA-Leu gene + intergenic spacer *trn*L-*trn*F)	trnLF_c	CGAAATCGGTAGACGCTACG	Taberlet et al. 1991	60	907-946
	trnLF_f	ATTTGAACTGGTGACACGAG	Taberlet et al. 1991		

Table S3. Additional *Piper arboricola*, *P. hancei*, and *P. kadsura* samples from GenBank were analyzed to explore the relationships among these three taxa within the complex. The scientific names, GenBank accession numbers, localities, submission years, relevant articles, submitting institutions, and voucher information of these additional samples are summarized below.

Taxon	GenBank accession #	Locality	Year	Relevant article	Submitter's institute	Voucher infromation
Piper arboricola C. DC.	AY572319	Viet Nam	2005	Direct Submission	Duke University, USA	MAJ 467
Piper arboricola C. DC.	JF491446	China	2011	Direct Submission	Fudan University, China	
Piper hancei Maxim.	EF450264	China	2008	Direct Submission	Fudan University, China	05120702 Y.Kang, Z.Chao (SHMU)
Piper hancei Maxim.	EF450265	China	2008	Direct Submission	Fudan University, China	05120703 Y.Kang, Z.Chao (SHMU)
Piper hancei Maxim.	EF450266	China	2008	Direct Submission	Fudan University, China	05120704 Y.Kang, Z.Chao (SHMU)
Piper hancei Maxim.	EF450267	China	2008	Direct Submission	Fudan University, China	06050901 Z.Y.Yang, W.B.Liu (SHMU)
Piper hancei Maxim.	EF450268	China	2008	Direct Submission	Fudan University, China	06050902 Z.Y.Yang, W.B.Liu (SHMU)
Piper hancei Maxim.	EF450269	China	2008	Direct Submission	Fudan University, China	06050903 Z.Y.Yang, W.B.Liu (SHMU)
Piper hancei Maxim.	EF450270	China	2008	Direct Submission	Fudan University, China	06050707 Z.Y.Yang, W.B.Liu (SHMU)
Piper hancei Maxim.	EF450271	China	2008	Direct Submission	Fudan University, China	06050708 Z.Y.Yang, W.B.Liu (SHMU)
Piper hancei Maxim.	EF450272	China	2008	Direct Submission	Fudan University, China	06110502 S.L.Pan, S.G.Ji (SHMU)
Piper hancei Maxim.	JF491444	China	2011	Direct Submission	Fudan University, China	
Piper hancei Maxim.	KF924137	China	2014	Direct Submission	Xinglong Tropical Botanical Garden, China	
Piper hancei Maxim.	HQ260457	China	2015	Direct Submission	Fudan University, China	
Piper hancei Maxim.	MZ636730	Viet Nam	2021	Direct Submission	Huế University, Viet Nam	
Piper hancei Maxim.	MZ636731	Viet Nam	2021	Direct Submission	Huế University, Viet Nam	

Piper hancei Maxim.	MZ602144	Viet Nam	2022	Direct Submission	Huế University, Viet Nam	
Piper hancei Maxim.	MZ602145	Viet Nam	2022	Direct Submission	Huế University, Viet Nam	
Piper kadsura (Choisy) Ohwi	DQ868724	Unknown	2008	Direct Submission	University of California, USA	
Piper kadsura (Choisy) Ohwi	EF450290	China	2008	Direct Submission	Fudan University, China	041001 S.L.Pan (SHMU)
Piper kadsura (Choisy) Ohwi	JF491439	China	2011	Direct Submission	Fudan University, China	
Piper kadsura (Choisy) Ohwi	KF924138	China	2014	Direct Submission	Xinglong Tropical Botanical Garden, China	
Piper kadsura (Choisy) Ohwi	MH493388	Japan	2018	Taxon 67 (4), 693-724 (2018)	University of Missouri St. Louis, USA	NT s.n.
Piper kadsura (Choisy) Ohwi	MH493389	Japan	2018	Taxon 67 (4), 693-724 (2018)	University of Missouri St. Louis, USA	NT s.n.
Piper kadsura (Choisy) Ohwi	MH493390	Japan	2018	Taxon 67 (4), 693-724 (2018)	University of Missouri St. Louis, USA	NT s.n.
Piper kadsura (Choisy) Ohwi	MH493391	Japan	2018	Taxon 67 (4), 693-724 (2018)	University of Missouri St. Louis, USA	NT s.n.
Piper kadsura (Choisy) Ohwi	MH493392	Japan	2018	Taxon 67 (4), 693-724 (2018)	University of Missouri St. Louis, USA	NT s.n.
Piper kadsura (Choisy) Ohwi	MH493393	Japan	2018	Taxon 67 (4), 693-724 (2018)	University of Missouri St. Louis, USA	NT s.n.
Piper kadsura (Choisy) Ohwi	OQ023331	China	2022	Direct Submission	Zhoushan Institute for Food and Drug control, China	
Piper kadsura (Choisy) Ohwi	OQ023332	China	2022	Direct Submission	Zhoushan Institute for Food and Drug control, China	
Piper kadsura (Choisy) Ohwi	OQ023333	China	2022	Direct Submission	Zhoushan Institute for Food and Drug control, China	
Piper kadsura (Choisy) Ohwi	OQ023334	China	2022	Direct Submission	Zhoushan Institute for Food and Drug control, China	
Piper kadsura (Choisy) Ohwi	OQ023335	China	2022	Direct Submission	Zhoushan Institute for Food and Drug control, China	
Piper kadsura (Choisy) Ohwi	OQ023336	China	2022	Direct Submission	Zhoushan Institute for Food and Drug control, China	
Piper kadsura (Choisy) Ohwi	OQ023337	China	2022	Direct Submission	Zhoushan Institute for Food and Drug control, China	
Piper kadsura (Choisy) Ohwi	OQ023338	China	2022	Direct Submission	Zhoushan Institute for Food and Drug control, China	

Piper kadsura (Choisy) Ohwi	OQ023340	China	2022	Direct Submission	Zhoushan Institute for Food and Drug control, China
Piper kadsura (Choisy) Ohwi	OQ023341	China	2022	Direct Submission	Zhoushan Institute for Food and Drug control, China
Piper kadsura (Choisy) Ohwi	OQ023342	China	2022	Direct Submission	Zhoushan Institute for Food and Drug control, China
Piper kadsura (Choisy) Ohwi	OQ023343	China	2022	Direct Submission	Zhoushan Institute for Food and Drug control, China
Piper kadsura (Choisy) Ohwi	OQ023344	China	2022	Direct Submission	Zhoushan Institute for Food and Drug control, China
Piper kadsura (Choisy) Ohwi	OQ023345	China	2022	Direct Submission	Zhoushan Institute for Food and Drug control, China
Piper kadsura (Choisy) Ohwi	OQ023346	China	2022	Direct Submission	Zhoushan Institute for Food and Drug control, China
Piper kadsura (Choisy) Ohwi	OQ023347	China	2022	Direct Submission	Zhoushan Institute for Food and Drug control, China

Table S4. The Shimodaira-Hasegawa (SH) and Kishino-Hasegawa (KH) tests indicate that (a) the *trn*L-F and ITS trees differ significantly in topology ($p < 0.05$), whereas (b) the Maximum likelihood (ML) and Bayesian inference (BI) trees do not show significant topological differences ($p > 0.05$).

(a)

	**–lnL**		**KH test (*p-value*)**	**SH test (*p-value*)**
*trn*L-F tree	11728.3365	(best tree)		
ITS tree	13900.0713		<0.0001*	0.0000*

(b)

	**–lnL**		**KH test (*p-value*)**	**KH test (*p-value*)**
ML tree	16509.35187	(best tree)		
BI tree	16511.47172		0.7057	0.3441

1 **Table S5.** (a) Morphological comparison between the descriptions of *Piper arboricola, P. kadsura, P. subglaucescens,* and *P. hancei*, indicating their conspecificity.

	***P. arboricola* C.DC.** (de Candolle 1920)	***P. subglaucescens* C.DC.** (de Candolle 1920)	***P. kadsura* (Choisy) Ohwi** (Ohwi 1965)	***P. kadsura* (Choisy) Ohwi** (Lin and Lu 1996)	***P. hancei* Maximowicz** (Tseng et al. 1999)	***P. kadsura* (Choisy) Ohwi** (This study)
Leaf shape	oblong-ovate, elliptic-lanceolate, 10–16 × 5–7 cm	oblong-elliptic, elliptic-lanceolate to lanceolate, 6–6.9 × 1.9–3.3 cm	ovate, 5–8 × 3–4 cm	ovate to oblong, 4–15 × 1–8 cm	ovate-lanceolate or elliptic, 6-12 × 2.5-4.5 cm	oblong, ovate, elliptic, ovate- elliptic, ovate-lanceolate, 5–8 × 2.5–4 cm
Leaf at apex	acuminate	sharply attenuate	long-acuminate	acute	acute or acuminate	acute or acuminate
Leaf at base	rounded, acute and equilateral	equal-sided, acute	rounded	cordate, rounded, obliquely or equilaterally obtuse to acute	gradually tapered, cuneate or rounded	cordate, cuneate or obtuse
Upper leaf surface	glabrous	-	dark green	-	glabrous	glabrous or puberulous

Lower leaf surface	sparsely hairy	-	paler beneath, and often sparsely covered with soft hairs	glabrous or puberulous	glabrous	glabrous or puberulous
Inflorescences	-	-	pendulous, 3-8 cm	erect	-	erect or pendulous
Male inflorescence	ca. 4 cm	ca. 4.2 cm	-	3–12 cm	6–10 cm	4–10 cm
Stamens	2, rarely 3	2	-	-	2	2 or 3
Female inflorescence	ca. 1.7 cm	ca. 2.5 cm	-	1–3.5 cm	ca. 3 cm	1.5–3.5 cm
Stigma	3	4	-	3-5	3-4	3-5
Fruits	ovate, glabrous	-	globose, 3–4 mm	globose, 3–4 mm	subglobose, 2.5–3 mm	globose, ca. 4 mm

Table S5. (b) Morphological comparison among the descriptions of *P. insectifugum, P. kwashoense*, and *P. philippinum*, indicating their distinctness.

	P. philippinum **Miq.** (Miquel 1843)	***P. insectifugum*** **C.DC.** (de Candolle 1869)	***P. kwashoense*** **Hayata** = *P. lanyuense* K.N. Kung & Kun C. Chang (Chang and Kung 2020)	***P. kwashoense*** **Hayata** (This study)
Petiole	1-1.5 cm	1.6 cm, very finely pubescent	1–4 cm, glabrous	1–4 cm, glabrous
Lamina	membranous	membranous	chartaceous, coriaceous	coriaceous
Leaf shape	elliptical, ovate, slightly obovate-elliptical, 10–10.5 × 3.5–4.5 cm, glabrous	round-ovate, 11.5 ×10 cm	ovate to cordate, 6–12 × 5–10 cm, glabrous	ovate-cordate, cordate, 10–15 × 8–12 cm, glabrous
Leaf at base	slightly unevenly rounded or rarely somewhat acute	equally rounded or somewhat truncate	cordate or truncate	cordate, rounded or truncate
Vein	5	9	5–7	5–9, when 9 veins, the

				outermost pair inconspicuous
Inflorescence	flexible (pendulous?)	erect	erect	erect
Male inflorescence	-	shorter than the leaves	4–10 cm	6–12 cm
Female inflorescence	10–12 cm	3–9 cm	3–7 cm	3–6 cm
Fruits	Ovoid, 4–5 mm	subglobose, 2–3 mm	subglobose, red, 4–5 mm	subglobose, red, 4–5 mm

Table S5. (c) Morphological comparison between the descriptions of *P. laosanum* and *P. sintenense*, indicating their conspecificity.

	***P. sintenense* Hatusima** (Hatusima 1935; Lin and Lu 1996)	***P. laosanum* C.DC.** (Suwanphakdee et al. 2023)	***P. laosanum* C.DC.** (This study)
Petiole	0.5–1 cm long, pilose or velutinous	0.2–1.5 cm long, pilose, hispid or puberulous	0.5–2 cm long, densely to sparsely hispid
Lamina	thin-chartaceous	chartaceous	chartaceous
Leaf shape	ovate-oblong, 5-14 cm × 1.8–6.5 cm	elliptic, ovate-subovate, lanceolate 6.6–12× 3.8–7 cm	ovate, lanceolate, ovate-lanceolate, 5–10 × 2–5 cm
Leaf at base	obliquely cordate, rounded or obtuse	oblique or slightly oblique	cordate, rounded or obtuse, slightly oblique
Upper leaf surface	hairy	hispid or pilose	densely to sparsely hispid
Lower leaf surface	hairy	puberulous or glabrous	densely to sparsely hispid
Inflorescence	pendulous	pendulous	pendulous
Male inflorescence	6–16 cm	5–16 cm	5–15 cm
Stamens	3	3–5	3–5

Female inflorescence	5–6 cm	4–7 cm	4–6 cm
Stigma	4 or 5, rarely 3	3–5 lobed	3–5 lobed

Table S5. (d) Morphological comparison between the descriptions of *P. kotoense* and *P. macropiper*, indicating their conspecificity.

	***P. macropiper* Pennant** (Quisumbing 1930)	***P. arborescens* Roxb.** = *P. kotoense* Yamam. (Yamamoto 1932)	***P. macropiper* Pennant** (This study)
Lamina	chartaceous	membranous to chartaceous	chartaceous
Leaf shape	lanceolate, 8–11 × 2–3.5 cm, glabrous	ovate-lanceolate or ovate, 9–13 × 3–6 cm, glabrous	ovate-lanceolate, ovate-elliptic or ovate, 8–12 × 3–5 cm, glabrous
Vein	5	5	5-7, when 7 veins, the outermost pair inconspicuous
Stigma	3	3	3
Fruits	fusiform, 3–4 mm in diameter	oblong, 1.5 mm long and 0.7 mm in diameter	sub-globose, 1–1.5 mm in diameter

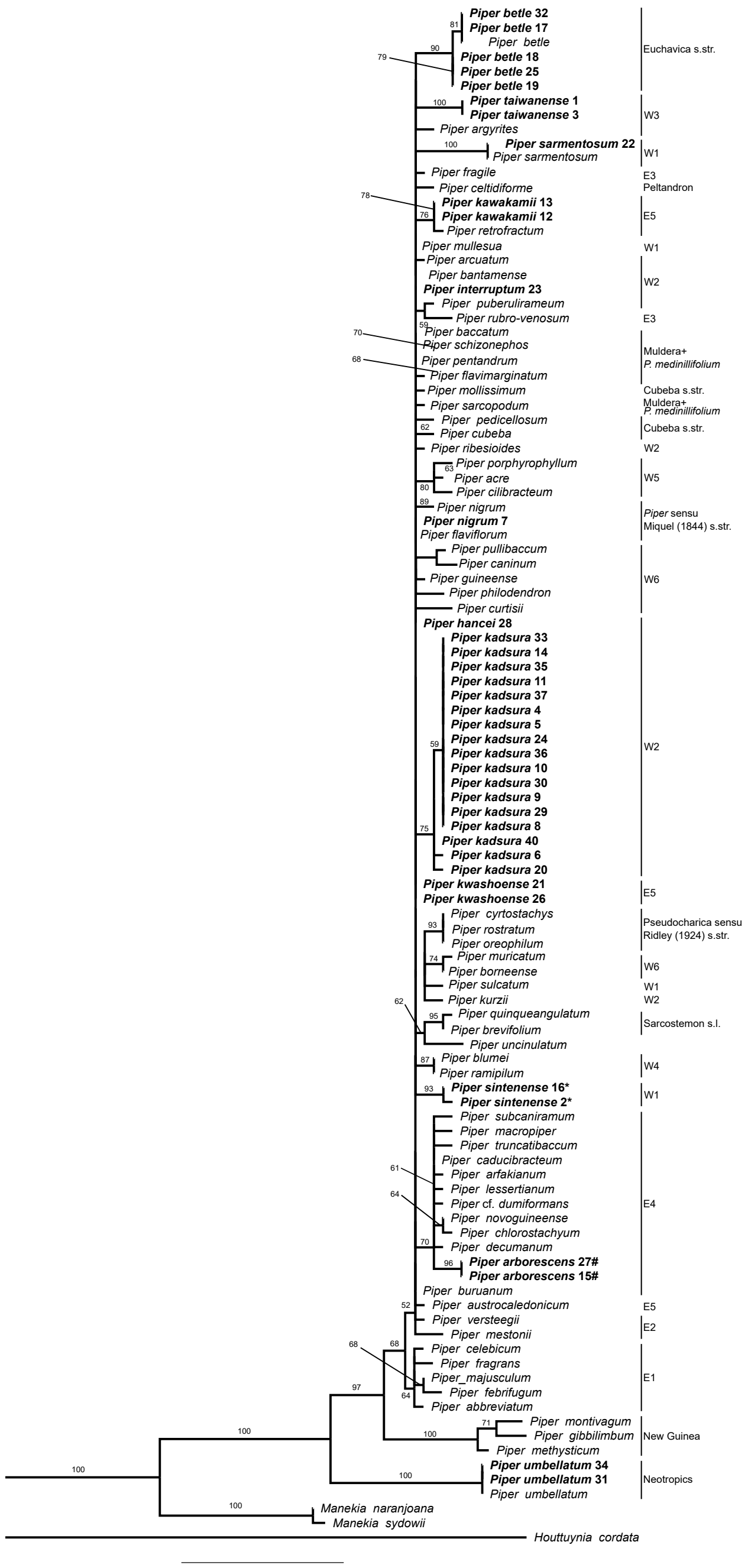

Fig. S1. Maximum likelihood tree based on *trn*L-F region. Vertical lines at the right denote the Asian clades. Numbers following scientific names correspond to the sample numbers listed in Table S1. Taxa marked with asterisks (*) and number signs (#) represent synonyms of *Piper laosanum* and *P. macropiper*, respectively. Bootstrap support values (>50%) are indicated at the corresponding branches. Newly generated sequences are shown in bold.

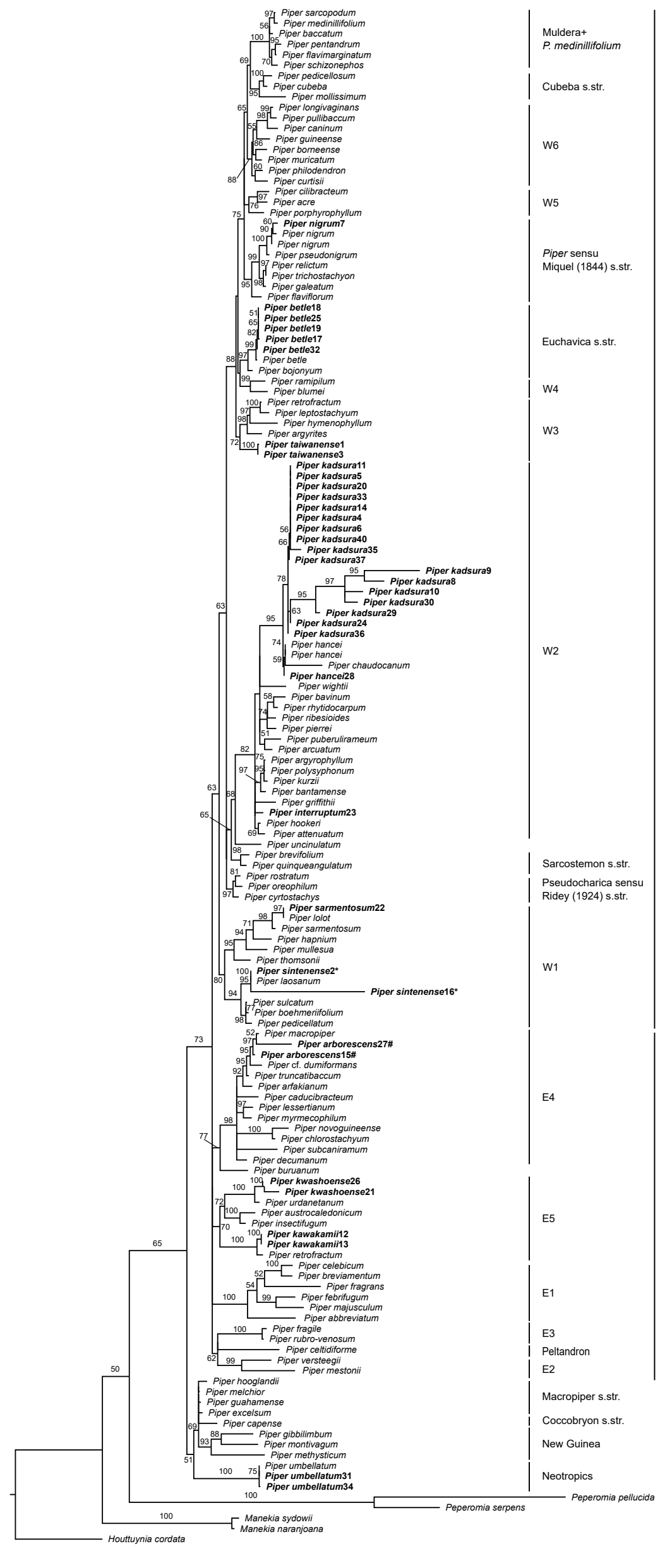

Fig. S2. Maximum likelihood tree based on the ITS region. Vertical lines at the right denote the Asian clades. Numbers following scientific names correspond to the sample numbers listed in Table S1. Taxa marked with asterisks (*) and number signs (#) represent synonyms of *Piper laosanum* and *P. macropiper*, respectively. Bootstrap support values (>50%) are indicated at the corresponding branches. Newly generated sequences are shown in bold. The crowns of clades W1–W6 and E1–E5 are labeled.
