# Supplementary material for: Phylogeny and taxonomic revisions of Piper (Piperaceae) in Taiwan
Source: J Plant Res. 2026 Aug 5;139(5):685–716. doi: 10.1007/s10265-026-01733-0 (PMC13588929; doi:10.1007/s10265-026-01733-0)
Supplement: Supplementary file 2 [file 10265_2026_1733_MOESM7_ESM.docx]

**Data S6**. The list of additional specimens examined in the present study.

*Piper betle*—

TAIWAN. Chiayi: Taiping, Kung et al. 2537 (CHIA); County Route 122 at 29K (Erpingzi), Kung et Ku 165 (CHIA); Zhonglun, Dapu Township, H.F. Yen 4800 (TNM). Pingtung: Kenting Forest Recreation Area, Kung et Ku 285, 286; Jiayi, Majia Township, Kung et Ku 207; Nanrenshan, K.N. Kung et al. 229, 234, 246 (CHIA); Gangkou, C.E. Chang s.n. (PPI); Kenting Park, Third Zone, S.Y. Lu 14058 (TAIF). Taitung: Lanyu Township, T.T. Lin 85, M.C. Ho 418, 423, Wang & Lai 6156, T.C. Huang 15717, Y. Yamamoto s.n. (NTUF), C.E. Chang 3608, 5615, 12435, 14490, 14991, 16670, 16672, 16720 (PPI), S.Y. Lu 17471, 17475, 17518 (TAIF); Yeyounanxi, F.L. Wang et al. 6409 (NTUF), Huang et Kao 5315 (TAI); Shiyiduishan, C.E. Chang 12123 (PPI); Langdao Village, C.E. Chang 11558, 11559 (PPI), T. Sata 1165 (TAI); Hongtoushan, C.E. Chang 16799, C.L. Yeh s.n. (PPI), Huang et Kao 6275 (TAI), Kung et al. 1030, 1036 (CHIA); Dasenshan, P.H. Chen 451 (PPI); Huandao Road, P.H. Chen 2205 (PPI); Qingsheshan, Kung & Hsieh 2567, 2569 (CHIA); Shantianshan, C.E. Chang 11608 (PPI); Damorshan, T. Sata 1172 (TAI); Yeyou Village, C.E. Chang 16729 (PPI), K.N. Kung et al. 581 (CHIA); Lanyu Township, Route East 80 at 27 km, Yang & Chuang 11427 (TNM); Xiaolanyu, Yeh & Yeh 97, 103 (PPI); Ludao Township, H.J. Su et al. 423 (NTUF), C.H. Chen et al. 7235 (TNM), C.E. Chang 16945, 16515 (PPI); Ring Road, Ludao Township, G.P. Hsieh 1424 (PPI); Nanliao–Huoshaoshan, Kudo et Mori 290 (TAI); Dulanshan, Donghe Township, C.S. Wu 15-007, 24-074; Tianchi, T.Y.A. Yang 9855 (TNM); Central Highway, Lanyu Township, Kung et al. 585, 1039, 1040, 2546, 2550; Ring Road, Lanyu Township, Kung et al. 1048, 2484, 2485, 2487, 2488, 2489, 2490; Yongxing Farm, Kung et al. 634, 637; Zhong'ai Bridge, Kung et al. 670, 1041; Ningpu, Changbin Township, Kung & Ku 320, 321, 323; Taiyuan Valley, Kung & Ku 329, 330, 333, 334, 340; Ruigang Highway, Kung & Ku 358, 360; Jhihben Forest Recreation Area, Kung & Ku 399, 415, 418; Lijia Trail, Kung et al. 819; Tulan, Dawu Township, Kung et al. 835 (CHIA).

*Piper kawakamii*—

TAIWAN. Kaohsiung: Beishoushan, K.N. Kung et al. 306, 307, 309, 310, K.N. Kung 851, 852, 853, Kung & Kung 707, 708, 709, 711, Kung & Hsieh 1443, 1444, 1445, 1446, 1447 (CHIA), C.S. Yang 28, 595 (TAIF). Pingtung: Sheding, Kung & Ku 210, 211, K.N. Kung et al. 220, 223, 224 (CHIA), H.J. Su 6144 (NTUF), L.W. Chang 19 (TAIF); Kenting National Park, S.Y. Lu 17291 (TAIF); Guijiaojiao, S.Y. Lu 14056 (HAST); Dajianshan, H.J. Su 3784 (NTUF); Chuanfanshi, Hukuyama 614; Gangkou, Manzhou Township, E. Matuda 2 (TAI); Eluanbi, N. Kung et al. 213, 215, 216 (CHIA), C.C. Chang 2239 (TAI); Hengchun, Fanzaoliao, Hibino et Suzuki 12503 (TAI); Kenting, C.C. Chang 2381, M.T. Kao 10107 (TAI), M.C. Ho 581 (NTUF); Sheding Park, J.C. Wang 2853; Guanshan, Huang et Huang 13506 (TAI); Qingwa Stone, P.F. Lu 4955; Chiniuling, S.Y. Lu s.n.; Xiangjiao Bay, K.C. Yang s.n. (TAIF); Kenting Forest Recreation Area, K.N. Kung et al. 273, 274, 275, 291, 292 (CHIA).

*Piper kwashoense*—

TAIWAN. Taitung: Lanyu, C.E. Chang 20009 (HAST), J.C. Wang 1520, 1611, 6826, T.C. Huang 6973, Y. L. Jeng 936, 1578, C.C. Hsu 4827, Huang & Ku 5315 (NTUF), C.E. Chang 12368, 12369 (PPI), Y.C. Jeng 1578, 1655, J.C. Wang 1520 (TAI), C.H. Ou et al. 37, 2308, Ou & Lu 9340 (TCF), S.Y. Lu 17595 (TAIF); Central Highway–Yeyin, K.N. Kung et al. 615 (CHIA); Datianchi, K.N. Kung et al. 629, 654, 1054, 1056 (CHIA), C.E. Chang 2244, 10257, 10257, 10302, 10302, 11369 (PPI), Huang & Hsu 4713 (TAI); Xiaotianshi, K.N. Kung et al. 642, 644 (CHIA), T.Y.A. Yang et al. 5934 (TNM), P.H. Chen 2217 (PPI); Circular Highway, K.N. Kung et al. 1049 (CHIA), P.F. Lu 6274 (TAIF), G.P. Hsieh 18610 (TNM); Zhongai Bridge, Y.L. Hsueh 12; Yeyin, Y.C. Li et al. 25, P.H. Chen 449 (PPI); Dongqing Beach, K.N. Kung et al. 630 (CHIA); Hongtoushan, K.N. Kung et al. 1038 (CHIA), C.E. Chang 16811, 22101 (PPI), T.Y.A. Yang et al. 2761 (TNM); Central Highway, K.N. Kung et al. 623 (CHIA), T.C. Huang et al. 9463 (NTUF), Huang & Yang 9463 (TAI), T.Y.A. Yang 3461, 4074 (TNM); Hongtou Nuclear Waste Site, K.N. Kung et al. 639 (CHIA); Dongqing River, C.E. Chang 10302; Shantianshan, C.E. Chang 11390; Qingshuishan, C.E. Chang 10257; Longmenshan, C.L. Yeh 591 (PPI); Lang Island, T.C. Huang et al. 9267 (TAI), T.Y.A. Yang 3102 (TNM), P.H. Chen 449 (PPI); Yeouyu Village, Liao & Chen 1172 (HAST); Yeouyu Nanxi, Wang & Lai 6408, 6409 (NTUF); Wangnan Peak, C.H. Ou 104 (TCF), T. Namba et al. 1878 (NTUF); Central Highway, Hongtou Village–Meteorological Station, Lu & Liao 1597, Y.C. Lu et al. 870 (PPI); Central Highway–Tianchi, C.K. Yang 299 (TNM); Hongtou Creek, C.C. Hsu 4990 (NTUF); Aobenshan, S.Y. Lu 25476 (TAIF); Longmen Port, C.H. Ou et al. 9340 (TCF). Ludao: Su & Huang 423, 424, T. Soma 611 (NTUF), C.E. Chang 15940, 16950, C.Y. Chiu 100 (PPI); Zhuangyuandi, K.N. Kung et al. 780, 781, 783 (CHIA); Guoshan Ancient Trail, K.N. Kung et al. 748, 750, 755 (CHIA), Hsieh & Hsieh 2083, G.P. Hsieh 1439, 1856 (PPI), C.H. Chen 7118, 7124, 7128, G.P. Hsieh 2080 (TNM), P.F. Lu 10829 (TAIF); Guanyindong, Kung & Hsieh 820 (CHIA), C.F. Chen 289 (PPI); Hot Spring Village, Hsieh & Hsieh 2126 (PPI).

*Piper laosanum* —

TAIWAN. Taipei: Houdong–Chailiao Ancient Trail–Zhongkeng Ancient Trail–Sandiaoling Waterfall, Kung & Hsieh 1509, 1510, 1512 (CHIA); Shuangxi Township, Dapingshan, S.C. Liu et al. 1208 (HAST), S.C. Liu et al. 1208 (TAIF); Wulai, C.K. Liou 660; Wulai, Badaoershan, Huang & Chang 66; Zhiliangjiushan, Y.H. Yu s.n.; Sanxia Township, Yunshen Waterfall, J.C. Wang et al. 8212; Hualien, Huagang Forest, H.Y. Chen 1192, H.Y. Chang et al. 1192; Bijiashan, S.Y. Lu 14458 (TAIF); Wulaishan, S.Y. Lu 17148, M.J. Lin 509, C.C. Chen 81 (TAIF), W.C. Leong 817 (HAST); Wulai Township, Xiaoyi, S.C. Liu et al. 281 (HAST), H.F. Yen 3694 (TNM), M.T. Kao 30054 (TAI); Wulai Township, Datongshan, P.F. Lu 9363 (HAST), C.M. Wang et al. 10864 (TNM), S.Y. Lu 16517 (TAIF); Wuzhishan, S.C. Wu et al. 2534 (HAST), S.Y. Lu 16517 (TAIF); Xindian, Wantan, H. Simizu 2305 (TAI); Fushan, S.T. Chiu 1025, 1028, 1029 (TNM), P.H. Lee 800 (TAIF); Sanxia Township, Yuanshan, C.H. Chen 5288; Wulai, Ayushan, Leu & Chen 1276 (TNM); Xindian, Wenshan Experimental Forest, K.Y. Wang 98; Pinglin, Bihu, C.H. Tsou et al. 437; Datunshan–Xishui, S.Y. Lu 15856 (HAST). Taoyuan: Longtan Township, Shimenshan, S.C. Liu et al. 699 (HAST), Liu & Chang 699; Beichatianshan, M.T. Kao s.n. (TNM). Hsinchu: Mawudu–Caiheshan, Y.Y. Huang 1240 (HAST), Y.Y. Huang 1240 (TAI); Wainiaozuishan, S.C. Wu et al. 2647 (HAST). Miaoli: Shitoushan, Tengping Ancient Trail, P.F. Lu 5544 (TAIF); Shitan Township, Xianshan, K.C. Yang s.n. (TNM). Taichung: Heping Township, Baimaoshan, C.I. Huang et al. 2488 (HAST); Guguan, C.H. Ou s.n. (TNM). Nantou: County Highway 151, K.N. Kung 854 (CHIA); Xitou, M.T. Kao s.n., C.M. Wang 4196 (TNM); Xitou, Sky Corridor, K.N. Kung et al. 968 (CHIA); Sun Moon Lake, Kudo & Sasaki 15600, T.I. Chuang 4873 (TAI); Sun Moon Lake, Zhuhu Trail, K.N. Kung et al. 985; Xitou, Streamside Trail, K.N. Kung et al. 961; to Sun-Link-Sea, Shewan, K.N. Kung et al. 973; Xinshan Forest Road, Kung & Chang 492 (CHIA); Yuchi Township, Lianhua Pond, Y.H. Chang 3845, S.Y. Lu 15306, 15478, 15870, Y.P. Cheng 2980 (TAIF), S.Y. Lu 15870, A. Huang s.n., Y.H. Lin s.n. (TNM); Ren’ai Township, Huisun Forest, Xiao Chushan Forest Road, C.C. Chen 631 (HAST); Renlun Forest Road, C.Y. Kuo 113, H.L. Chiang 2475 (TAIF); Xinyi Township, Shenmu Village, Chen & Wang 5784 (TNM); Lugu, P.H. Chen 696 (PPI). Chiayi: Ruitai Ancient Trail, Kung & Ku 157 (CHIA); Shimeng Valley, C.Y. Liang 1119 (CHIA); Alishan Township, Shizilu, H.F. Yen 11170 (TNM). Tainan: Xinhua, H.F. Yen 224 (TNM). Kaohsiung: Maolin Township, Shanping, J.C. Wang et al. 9666, 6510 (HAST), S.Y. Lu s.n., 16106, 16127; Duona, S.Y. Lu 24452 (TAIF); Maolin Township, Shanping–Tengzhi, J.C. Wang et al. 8212, 9666, S.Y. Lu 16127 (TAIF); Taoyuan Township, Zhongzhiguan Ancient Trail, Kung & Chang 482 (CHIA); Nanhui Highway, C.S. Kuoh 15498 (NCKU); Tengzhi, H.P. Tseng 93 (PPI); Wangzishan–Minghaishan, P.H. Chen 3914 (TAIF). Pingtung: Sheting, Kung & Ku 242, 243; Nanrenshan, K.N. Kung et al. 244 (CHIA); Zhenlisan, G.P. Hsieh 984, 1028, 1401, 2547, K.P. Lo 280, S.M. Ko 1243, J.F. Chen 92 (PPI), S.M. Ku 1243 (TAIF), T.Y.A. Yang et al. 16507, 17210 (TNM); Lilongshan, C.F. Chen 2005, G.P. Hsieh 1028, C.L. Yeh s.n.; Yidingshan, C.F. Chen 969, K.P. Lo 576 (PPI); Guitian, S.Y. Lu 14081 (TAIF). Taitung: Dahu, Taiwan Keteleeria Forest Reserve, K.N. Kung et al. 739 (CHIA); Dahu, S.Y. Lu 14100, H.S. You 14134 (TAIF). Yilan: Quantoumu Trail, Kung & Hsieh 1476, 1478, 1483 (CHIA); Jiaoxi Township, Taroko National Park, W.C. Leong et al. 3540 (HAST); Fushan, P.H. Lee 800, C.C. Hsu s.n. 248, 420 (TAIF), T.Y.A. Yang 3600 (TNM); Fushan Botanical Garden, K.C. Chang 4795, 4783 (TAIF), T.Y.A. Yang 11949 (TNM); Sanxing Township, Qingshui, Y. Simada 5891 (HAST).

*Piper macropiper* —

TAIWAN. Taitung: Lanyu, C.H. Chang 3655, C.C. Hsu 9492, T.C. Huang 9210, Y.C. Jeng 1609, C.E. Chang 3655, 16905, S. Sasaki 14, Huang et al. 9420 (TAI), S.Y. Lu 3673, 18431 (TAIF), T.T. Lin 84, Ho & Huang 420, 421 (NTUF), C.E. Chang 16660, 16661, 16662, 16663, 16664, 11752 (PPI); Tianchi, K.N. Kung et al. 23, 49, 51, 53, 651, 656, 658, 652, 678, 646, 672 (CHIA), S.F. Huang 2688, Huang & Yang s.n., Wang & Lai 6238 (NTUF), Huang & Hsu 4719 (TAI); Zhongai Bridge, J.C. Wang 6000 (PPI); Daisenshan, T. Sata 1169 (TAI); P.H. Chen 450, 583 (PPI); Langdao Village, T.Y.A. Yang 3102 (TNM); Langdao, Wang & Lai 6146 (NTUF); Hongtou Village, T.C. Huang et al. 9210, C.C. Hsu 9492; Hongtou–Tianchi, S.F. Huang 2688; Hongtoushan, K.N. Kung et al. 1031, 1037, 1033, 1034, 2477 (CHIA), C.E. Chang 17790, 19217-1, 19217-2, 19217-3, 19217-4 (PPI), T.Y.A. Yang 2761 (TNM); Qingsheshan, Kung & Hsieh 2575, 2577 (CHIA).

*Piper kadsura* —

TAIWAN. Taipei: Yangmingshan, J.C. Liao 10816 (NTUF), C.K. Liou 204 (TAIF); Shuangxi, C.E. Chang 16713 (PPI); Hapen, S.Z. Yang 27647 (PPI); Xindian, Wantan, H. Simizu 2155; Thumbshan, Suzuki & Kohasikawa 4327; Wulai, C.C. Hsu s.n.; Wanli, C.M. Kuo 5355; Neishuangxi, C.T. Wamg 1000; Sanxia, J.C. Wang 3260; Aodi, S.F. Huang 2897 (TAI); Menghuan Lake, R.G. Shi 40 (TAI), H.L. Chiang 385 (TAIF); Zhuzihu, S.Y. Lu 24395; Tamsui, W.F. Ho 1405; Daman, S.Y. Lu s.n.; Caoling Historic Trail, C.K. Liou 184, H.L. Chiang 167; Datunshan, T.T. Chen 8234; Shimen, W.F. Ho 256; Huanggangshan, J.H. Lii 338; Manyueyuan, C.L. Huang s.n.; Shifen Waterfall, Pingxi, C.C. Chen 719 (TAIF); Gongliao Township, Fulong, Chen & Sang 767; Ruifang Township, Shumeiping, T.Y.A. Yang et al. 5549 (TNM); Provincial Highway 2, 116K, Kung & Hsieh 1484; County Highway 102A, Kung & Hsieh 1488, 1495; Houdong Highway, Kung & Hsieh 1496, 1500; Houdong–Chailiao Historic Trail, Kung & Hsieh 1502, 1507 (CHIA); Huangzuishan, P.H. Chen 1246 (TAIF). Taoyuan: Baleng, S.Y. Lu 19134; Daxi Township, Shigui Pit, C.C. Chen 428 (TAIF); Daman–Beiheng Highway, Lin & Chen 1 (TNM). Taichung: Baxianshan, C.C. Kao 508 (NTUF); Huisun Forest, N. Ueyama 38 (TAI), Kung & Hsieh 20 (CHIA); Dongshi, S. Suzuki s.n., C.M. Kuo 7542; Bailu, M.T. Kao 9668; Baileng–Dongmaoshan, W.S. Tang 352 (TAI); Dakeng Trail, Y.H. Tseng 467 (TCF), W.F. Ho 1013 (TAIF); Anmashan, W.F. Ho 1426; Jiabao Terrace, S.Y. Lu 19098 (TAIF); Wushe Pit, Heping Township, C.M. Wang 4114; Guguan, C.H. Ou s.n.; Daxueshan 200 Forest Road, Heping Township, C.M. Wang 4468 (TNM); Henglingshan, P.H. Chen 1976 (PPI). Hsinchu: Qingshi, Jianshi Township, T. Yu 16353 (NCKU); Naluo–Shang Yuliao, C.M. Wang 2168 (TNM); Guanxi, H.S. Liau 411; Wuzhishan, Huang & Huang 15159 (TAI). Miaoli: Dahu, Y.C. Chen 111; Taian Hot Spring–Hushan, Jang & Ma 90; Shuijing, Sanyi Township, C.H. Yu 487 (TAI); Fayun Temple, Dahu Township, C.M. Wang 5431; Guandaoshan, C.F. Chen 5553; Duchangkou, Nanzhuang Township, C.M. Wang 5153; Xianshan, Shitan Township, K.C. Yang et al. 4399 (TNM); Miaoli: S.H. Liu 2180 (TAIF). Nantou: Xitou, T.Y. Lin 140 (TNM), K.N. Kung et al. 964, 969, 970 (CHIA); Phoenixshan, Xitou, F.L. Wang 6551 (NTUF), K.N. Kung 865 (CHIA); Heshe, T.C. Huang 2128; Qingshuigoushan, T.C. Huang 5291; Mingjian–Ershui, S.F. Huang 4489 (TAI); Guandao Stream, Lu & Ou 4565 (TCF), W.F. Ho 181 (TAIF); Sun Moon Lake, S.Y. Lu 18732 (TAIF), K.N. Kung et al. 983 (CHIA); Guangaoshan, H.L. Chiang s.n.; Ruiyan Stream, Y.P. Cheng 3286 (TAIF); Aowanda, Ren’ai Township, C.H. Chen 5288; Bihu, S.T. Chiu 1743 (TNM); Lianhuachi, Yuchi Township, Kung & Chang 491, 494; Dongdingshan, Kung & Chang 502, 508; County Highway 151, K.N. Kung 857 (CHIA); Wushe, P.H. Chen 785, 786 (PPI). Changhua: Zhuxing Pit, Fenyuan Township, C.Y. Tsai 4055 (TNM). Yunlin: Huben, Linnei Township, Y.H. Tseng 2437 (TCF). Chiayi: Jiali Ping–Ruili, K.C. Yang 3526 (TAI); Dongzijiao, Lu & Ou 373 (TCF); Caoshan, Fanlu Township, W.P. Leu s.n.; Nanxi Forest Road, K.C. Tang 5113 (TNM); Datongshan, Wang & Chang W00520 (TNM), K.N. Kung 929 (CHIA); Longyinshan, K.N. Kung 8; County Highway 169, 9K, K.N. Kung 68, 69 (CHIA); Ruitai Historic Trail, Kung & Ku 158, 160, 176, 180, 194; Shilong Forest Farm, Kung & Kuo 703; Shanziding Botanical Garden, Kung & Chang 905 (CHIA). Tainan: Xinhua Forest Farm, S.Z. Yang 1127 (PPI); Guanziling, Morimoto 556 (TAI); Kantoushan Trail, Kung & Lu 61, 64 (CHIA). Kaohsiung: Southern Cross-Island Highway, C.S. Kuoh 12367 (NCKU); Jiaxian, S.Z. Yang 2653 (PPI); Xinanshan–Tengzhi, Yamamoto & Mori 747; Neimen–Muzha, S. Suzuki 5947; Liugui, J.M. Chao 675 (TAI); Shanping, C.T. Moo 2166 (TAI), C.C. Chen 587 (TAIF); Beishoushan, C.S. Yang 680 (TAIF), P.H. Chen 1353 (TAIF), K.N. Kung et al. 303, 315 (CHIA); Fenggang Forest Road, H.L. Chiang 1275 (TAIF); Maolin, H.L. Chiang 2022; Colorful Butterfly Valley, Liugui Township, H.F. Yen 5035 (TNM); Taoyuan Township, Kung & Chang 445 (CHIA). Pingtung: Jialeshui, C.H. Su 160 (NTUF); Taiwu, C.F. Chen 1853; Zhenlishan, G.P. Hsieh 100, 2575; Kasan, C.F. Chen 521; Xiaoliuqiu, G.P. Hsieh 1552, P.N. Wang 16715; Kenting, Sheting, C.E. Chang et al. 22139; Lilongshan, S.Z. Yang 30621, P.H. Chen 203 (PPI); Yidingshan, G.P. Hsieh 1066; Liuqiu Islet, T.Y. Lo 40; Wutoushan, H.P. Tzeng 167; Jinshuiying, S.Z. Yang 30592 (PPI); Xiaoliuqiu, T. Hosokawa 1669, S.F. Huang s.n. (TAI); Xiaoliuqiu, Meirendong, P.H. Chen 659, 660 (PPI); Eluanbi, Kudo & Suzuki 15727 (TAI), W.L. Chiou 11106 (TAIF); Nanrenshan, T.C. Huang 9556 (TAI), C.H. Ou 7628 (TCF), T.Y.A. Yang 2591 (TNM), K.N. Kung et al. 227, 231, 271, P.H. Chen 536 (PPI); Xiangjiao Bay, Huang & Hunag 8357 (TAI); S.Y. Lu 13897 (TAIF); Guanshan, Hengchun, S.Y. Lu 19205; Beidawushan, S.Y. Lu 14152 (TAIF); Sheting Park, K.N. Kung et al. 209; Kenting Forest Recreation Area, K.N. Kung et al. 293 (CHIA). Taitung: Daren, K.P. Lo 1038; Zhiben, C.E. Chang 2583; Ludao Township, C.E. Chang 16718 (PPI); Taiwan Walnut Conservation Area, S.Z. Yang 30156, 30336 (PPI), S.Y. Lu 18392 (TAIF), S. Saito 142 (TNM); Xinwu Beach Palm Conservation Area, S.Z. Yang 26739 (PPI), Liu & Keng 2779 (TAI); Xinwu, S.Y. Lu 18333; Wulu, K.Y. Li 843; Xinkangshan, S.Y. Lu 19347; Daananshan, Liu & Chen 769 (TAIF); Luoshan, Kung & Ku 341, 348; Sixty Stoneshan, Kung & Ku 350; Dulanshan, Kung & Ku 371, 380; Zhiben Forest Recreation Area, Kung & Ku 400, 414; Dawu Taiwan Yew Conservation Area, Kung & Hsieh 725, 736 (CHIA). Hualien: Xiulin, T. Suzuki et al. 17681; Yuli, Suzuki–Tokio 19848; Lanshan, C.C. Hsu 3700 (TAI); Xiulin Township, Dali, Z.W. Lee et al. 592 (TAIF); Xiulin Township, Heping Forest Road, S.M. Liu et al. 349, S.T. Chiu et al. 6127 (TNM); Lushui, T.T. Lin 40; Huiyuan, S.Y. Lu 15893; Taroko, S.Y. Lu 15893 (TAIF); Nan’an, Walami Trail, Kung & Hsieh 27, 38, C.Y. Liang 689; Ruigang Highway, Kung & Ku 368 (CHIA). Yilan: Datong Township, Nanshan–Siyuan, C.M. Wang 2306 (PPI), C.M. Wang 2468 (TNM); Taipingshan, S. Suzuki s.n. (TAI), S.Y. Lu 14274, C.K. Liou 477 (TAIF); Nanao, Shenmi Lake, Huang & Huang 13884 (TAI); Fushan, C.K. Liou 249 (TAIF); L.F. Chang 958, 959 (CHIA), Z. Debreczy et al. 49250 (TAIF); Toucheng Township, Guishan Island, C.F. Chen 3236 (TNM); Qilan 100 Forest Road, Kung & Hsieh 1457, 1461; Beiheng, Kung & Hsieh 1462; Taiping Forest Road, Kung & Hsieh 1466, 1471; Songluo Lake Trail, Kung & Hsieh 1472, 1475; Qiantoumu Trail, Kung & Hsieh 1479, 1480 (CHIA).

*Piper taiwanense*—

TAIWAN. Taoyuan: Shimen Reservoir, P.H. Chen 1242 (TAIF). Miaoli: Zhuolan, Changqing Valley, Huang et al. 15605 (TAI); Shitan Township, Beiwuo, C.H. Chen 4127 (TNM). Nantou: Yuchi Township, Lianhua Pond, Xinshan Forest Road 1K, Kung & Chang 484, 487 (CHIA); Lugu, Dongdingshan, Y.L. Hsueh 511; Lianhua Pond, Yan’s Ranch 1 km Signpost, K.N. Kung et al. 981 (CHIA); Lianhua Pond, K.N. Kung et al. 978, 981, 1450, 1456 (CHIA), M.T. Kao 4058 (TAI); Sun Moon Lake, Kudo & Sasaki 15328; Guangfu Township, Dafu, Y. Yamamoto 2961 (TAI); Jiji, S.Y. Lu 24734 (Paratype) (TAIF). Chiayi: Shinnong Forest, Kung & Kuo 700 (CHIA). Tainan: Dongshan Township, Kantoushan Trail, Y.L. Hsueh 510 (CHIA). Kaohsiung: Beishoushan, K.N. Kung 713; Taoyuan Township, Zhongzhiguan Ancient Trail, Kung & Chang 479 (CHIA); Tengzhi, P.H. Chen 1277 (TAIF). Pingtung: Mudan, C.E. Chang 4749; Laofoshan, G.P. Hsieh 1381 (PPI); Gaoshi, Kudo & Suzuki 16018 (TAI), T.T. Chen 832 (TAIF); Zhenlisan, G.P. Hsieh 1401 (TNM); Laisheshan, P.H. Chen 2242 (PPI); Liangshan, P.H. Chen 141 (PPI). Taitung: Donghe Township, Dulanshan, C.S. Wu 18-009 (TNM). Hualien: Lintianshan, C.E. Chang 27230 (PPI). Yilan: Taipingshan, S. Suzuki 4097 (TAI).

*Piper umbellatum* —

TAIWAN. Chiayi: Pinglin, M.T. Kao 10352 (TAI); Dongzijiao, Lu & Ou 381 (TCF); Alishan Township, Caoshan, H.F. Yen 4751 (TNM). Tainan: Pinglin, C.S. Kuoh 10937 (NCKU); Nanhua, Y.H. Tzeng 93 (TAIF). Kaohsiung: Shanping Forest Road, Lai & Peng 126 (TNM), J.H. Lii 757 (TAIF); Taoyuan Township, Baolai, C.M. Wang W00795; Liougui, C.H. Liou 1510 (TAIF); Jiasian Township, Baiyunshan, H.F. Yen 2665; Taoyuan, C.M. Wang W00795 (TNM). Pingtung: Manzhou Township, Nanrenshan, Lu & Chen 1402, T.G. Lammers 8491 (HAST), M.T. Kao 7339, R.T. Li 3219 (TAI), Y.S. Tseng 10112 (TCF), T.Y.A. Y. et al. 14878 (TNM); Lilangshan, J.F. Chen 149 (PPI); Gaoshi, Kudo & Suzuki 15922; Shouka–Chushui Branch, Liu & Keng 2810; Guizijiao, Kawakami & Sasaki s.n.; Gaoshi, Kudo & Suzuki s.n. (TAI); Changle Village, S.W. Chung et al. 7319 (TAIF). Taitung: Dahu Township, Taiwan Yushan Conservation Area, K.N. Kung et al. 726; Dahu Township, Tushan, K.N. Kung et al. 742 (CHIA); Hongye Village, T.Y.A. Yang et al. 5930 (PPI), T.Y.A. Yang 5930 (TNM); Biru, H.F. Yen 4908 (TNM); Zhiben, S.Y. Lu 8386 (TAIF); Dulanshan, C.S. Wu 14-026; Guitian, Yang & Hsu 1163 (TNM).
